# Microwave-Assisted Deoxygenation of Substituted Aromatic Ketones over Commercial Pd/Al_2_O_3_ under Mild Conditions

**DOI:** 10.1021/acsomega.5c12401

**Published:** 2026-03-12

**Authors:** Fabio Bucciol, Ignacio C. Vega, Emanuela Calcio Gaudino, Maela Manzoli, Silvia Tabasso, Giancarlo Cravotto

**Affiliations:** Department of Drug Science and Technology, 9314 University of Turin, Via Pietro Giuria 9, Turin 10125, Italy

## Abstract

Herein, a study on the deoxygenation of substituted aromatic ketones over commercial Pd/Al_2_O_3_ (5 wt %) is reported. The reaction occurs under very mild conditions (120 °C, 5 bar H_2_) in just 2 h using microwaves to enhance the kinetics. Ethanol was found to be the best solvent, while the effect of the substituents was studied over different substrates. On the basis of experimental evidence, a mechanism is proposed in which keto–enol tautomerization enables deoxygenation to the corresponding alcohol followed by direct hydrogenolysis to the alkyl chain.

## Graphical abstract

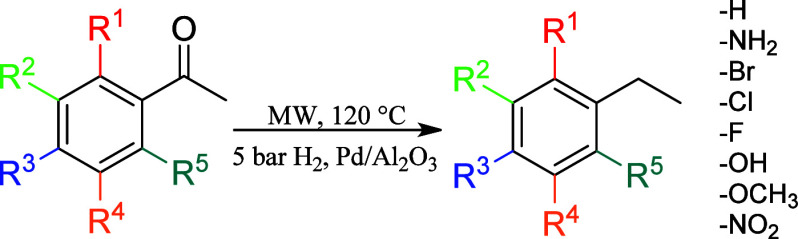

## Introduction

Deoxygenation reactions represent a fundamental transformation in organic synthesis^1–3^ and are gaining increasing interest due to the growing emphasis on biomass-derived chemicals. Biomass conversion processes often yield substituted oxygenated intermediates containing carbonyl groups,^4–6^ which can be further reduced to obtain products similar to those derived from fossil resources, with a view to developing a biorefinery.^7^ Reliable and mild deoxygenation methods are therefore crucial to facilitate the transformation of these renewable resources into valuable products. The reduction of ketones to alcohols and alkanes finds applications ranging from fuels to drug synthesis^8,9^ ; however, it is limited by the intrinsic stability of their carbonyl group. Most of the works in the literature deal with the hydrogenation of ketones to form the corresponding alcohols,^10,11^ but reports on the complete deoxygenation to alkanes are quite limited. A traditional approach is the Clemmensen reduction,^12–14^ which, however, despite its proven results, relies on hazardous reagents and harsh conditions. A recent paper reported a Clemmensen reaction in mechanochemical conditions replacing the amalgam with Zn powder and concentrated HCl with hydrated *p*-toluenesulfonic acid.^15^ An alternative approach is the reduction of ketones using noble metal catalysts, often through a hydrogen transfer mechanism.^16–19^ Even though this reaction proceeds under mild conditions, the need for an excess of donating reagent introduces inefficiencies and additional wastes. Pino and co-workers investigated the mechanism of acetophenone reduction over Pt and Co supported over SiO_2_, using molecular hydrogen.^20^ Their work highlighted the synergy between the two metals of the bimetallic catalyst in tuning the selectivity; however, the reaction conditions remained harsh (up to 240 °C and 40 bar of H_2_)_._ Saraeian and co-workers also studied acetophenone deoxygenation in similar conditions but focusing on Pd bimetallic catalysts.^21^ Near complete selectivity to ethylbenzene was observed using Pd/Fe (0.8 wt % of Pd), speculating that the result is a combined effect of modification of Pd electronic properties in the presence of Fe and the unfavorable ring hydrogenation due to its lower binding energy over the Pd/Fe surface.

The electronic properties of supported Pd were also studied by Kim et al., preparing Pd/Al_2_O_3_ and Pd/SiO_2_ via double flame spray pyrolysis.^22^ Indeed, acidic supports can produce ionic effects on metal surfaces and affect the surface electronic properties, thus favoring the dissociation of hydrogen.^23^ In that study, a complete conversion was observed at 15 °C with just 3 bar of H_2_; nevertheless, the reaction mostly produced phenylethanol, while ethylbenzene was only observed with an 11.8% yield. Substituents on the aromatic ring can strongly influence the yield. For example, Goclik and co-workers investigated the hydrodeoxygenation of substituted hydroxyacetophenones over an FeRu catalyst immobilized on an imidazolium-based supported ionic liquid phase (SILP) at 175 °C and with 50 bar of H_2_.^24^ However, 16 h was required to achieve almost complete conversion.

In this context, we report a straightforward deoxygenation of substituted aromatic ketones over a commercial Pd/Al_2_O_3_ (5 wt %) catalyst under minimal H_2_ pressure and mild conditions (Scheme 1). A deep investigation of solvent choice and substrates over the reaction yield is provided. Also, a reaction mechanism is proposed. To further increase the efficiency of the reaction, microwaves (MWs) were used to enhance the kinetics, thus shortening the reaction time. The ability of MWs to promote the reactivity over metallic heterogeneous catalysts is well-known in the literature^25,26^ and has already been investigated in our previous work on reductive amination.^27^

**Scheme 1:**
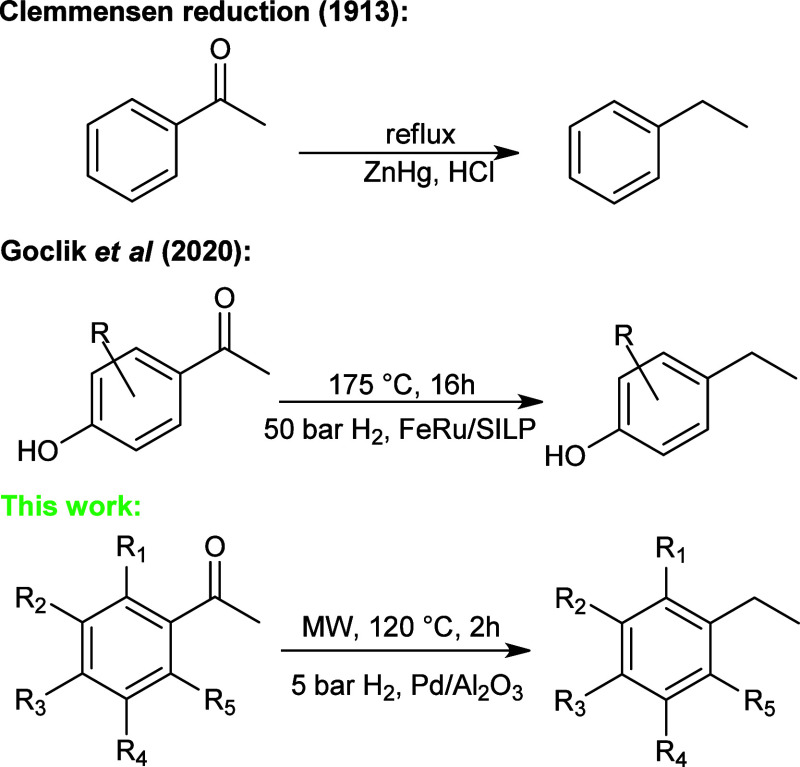
Reduction of Aromatic Ketones

## Results and Discussion

### Screening of Reaction Conditions

A preliminary screening of different solvents was performed for the deoxygenation of 4-hydroxyacetophenone (4-HAP) as a benchmark reaction over commercial Pd/Al_2_O_3_ at 120 °C under 5 bar of H_2_. First, a screening of solvents of different polarities was performed. As shown in Table 1, the use of alcohols as solvents improved both conversion and selectivity, with quantitative values being achieved in the presence of ethanol (EtOH), 2-propanol (i-PrOH), 1-propanol (n-PrOH), and 1-butanol (n-BuOH). Quantitative conversion was achieved also with ethyl acetate (EtOAc) and cyclopentyl methyl ether (CPME); however, the hydrogenation of the aromatic ring was observed, leading to the formation of byproducts such as 4-ethylcyclohexan-1-one and 4-acetylcyclohexan-1-one.

**Table 1:** Screening of Solvents for the Deoxygenation of 4-HAP to 4-Ethylphenol^b^

**entry**	**solvent**	**conv.** ^a^ **(%)**	**sel.** ^a^ **(%)**	**yield** ^a^ **(%)**
1		60	61	36
2	EtOAc	100	60	60
3	CPME	100	82	82
4	MeOH	69.8	82	57
5	EtOH	100	100	100
6	n-PrOH	100	100	100
7	*i*-PrOH	100	100	100
8	*n*-BuOH	100	100	100

a GC-MS integrated areas %.

b Reaction conditions: solvent (1 mL), 4-HAP (2 mmol), catalyst/substrate 1:1000 mol_Pd_/mol, H_2_ (5 bar), 120 °C, 2 h.

A screening of the effect of temperature and catalyst amount was performed using EtOH as a solvent. As shown in Table 2, the conversion of 4-HAF was already complete at 80 °C (Table 2, entry 2); however, below 120 °C, reductive etherification between 4-HAF and EtOH was observed due to a slower or incomplete hydrogenation rate of the carbonyl group, thus lowering the selectivity. Conversion was also affected by the catalyst amount since halving it (Table 2, entry 5) reduced it to 45%. Overall, temperature mostly influences the selectivity, while the catalyst amount influences the conversion. To investigate the possible participation of the solvent in the hydrogenation reaction, a test using 2-propanol under a N_2_ atmosphere was performed. However, no conversion was detected, thus proving that this solvent did not exert any hydrogenating effect when used alone in the presence of Pd/Al_2_O_3_ (Table 2, entry 7).

**Table 2:** Screening of the Reaction Conditions for the Deoxygenation of 4-HAP to 4-Ethylphenol^d^

**entry**	***T* (°C)**	**cat/sub (mol**_**Pd**_ **/mol)**	**conv.** ^a^ **(%)**	**sel.** ^a^ **(%)**	**yield** ^a^ **(%)**
1	60	1:1000	69	59	41
2	80	1:1000	100	51	51
3	100	1:1000	100	64	64
4	120	1:1000	100	100	100
5	120	1:2000	45	100	45
6^b^	120	1:1000	n.d.		
7^c^	120	1:1000	n.d.		

a GC-MS integrated areas %.

b Reaction under a N_2_ atmosphere.

c Reaction under a N_2_ atmosphere and *i*-PrOH as solvent.

d Reaction conditions: EtOH (1 mL), 4-HAP (2 mmol), H_2_ (5 bar), 2h.

### Catalyst Recycling

ICP analysis of the commercial Pd/Al_2_O_3_ showed that the palladium loading is close to the nominal one (5 wt %). Moreover, the alumina support is mainly in the cubic phase (00–002–1421) but also peaks in positions compatible with the hexagonal (00–002–0921) and rhombohedral (00–002–1227) phases were observed (Figure S1a). No peaks due to crystalline Pd have been detected, likely indicating a high metal dispersion. This is confirmed by FESEM measurements, which revealed the presence of Pd nanoparticle agglomerates accompanied by Pd nanoparticles with mean diameter *d*_m_= 5.6 ± 1.1 nm (Figure S1b–d).

The recyclability of the catalyst was then tested under the optimized conditions, using 4-HAF as the substrate.

As shown in Figure 1, the catalyst retains its activity for at least three cycles, as far as conversion is considered. However, from the third catalytic cycle, selectivity drops because of the reductive etherification of the ketone toward its ethyl ether product (Figure 1a). Since it was demonstrated that also other alcohols favor the reaction (Table 1), EtOH was replaced by i-PrOH for the next two cycles (Figure 1b). The idea was that a more hindered alcohol would be less likely to attach to the carbonyl by reductive etherification, thus enhancing the selectivity toward the desired product. Indeed, an increase of the selectivity toward 4-ethylphenol was obtained with 88% selectivity still after 5 cycles. This proves that the catalyst is stable over five reaction cycles, likely indicating that highly dispersed Pd species are stable and no coalescence phenomena occurred under reaction conditions.

**Figure 1:**
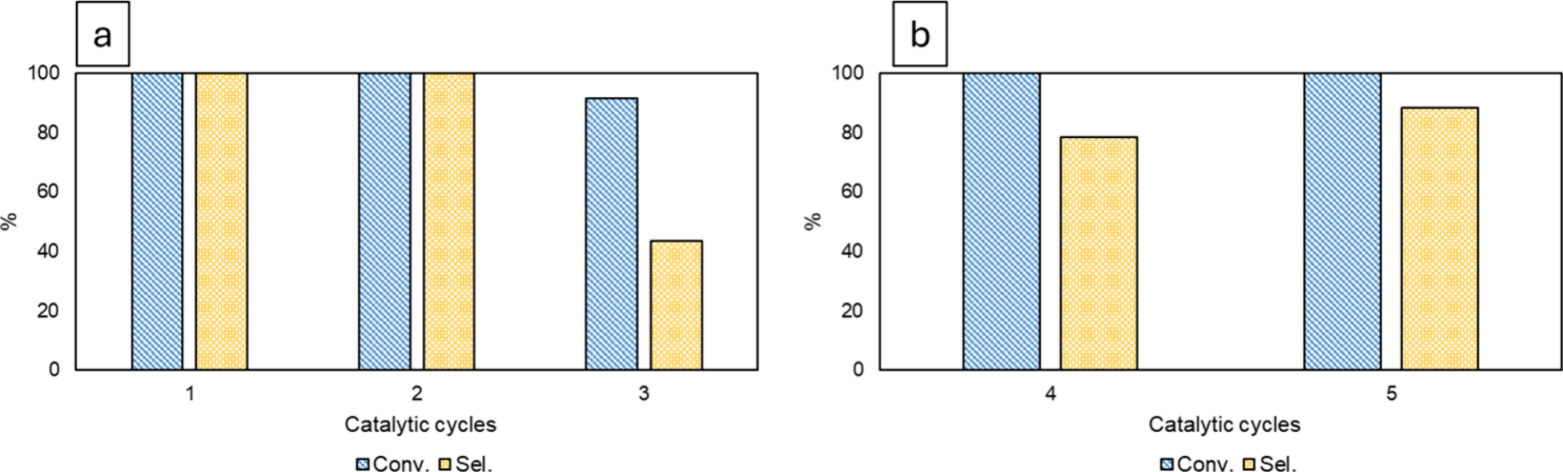
Conversion and selectivity for deoxygenation of 4-HAF over five catalytic cycles. (a) EtOH and (b) i-PrOH.

However, ICP analysis showed some leaching (26%) after the fifth run, which suggests that after five cycles the catalyst lost activity due to metal leaching, which was not observed after the first run.

### Reaction Mechanism Hypothesis

The first step in understanding the mechanism is to establish whether alcohol also acts as a hydrogen donor. Therefore, the reaction was performed under the optimized conditions, but without molecular H_2_ (Table 2, entries 6 and 7). Using 4-HAP as the substrate, no conversion was observed with either EtOH or i-PrOH (which is a common hydrogen donor), while a 36% yield for the deoxygenated product was found using H_2_ in solventless conditions (Table 1, entry 1). These findings rule out the hydrogen transfer mechanism.

It could be speculated that the reaction involves two steps with the alcohol as an intermediate (Scheme 2). The hydrogenation from ketone (**1**) to alcohol (**3**) is likely to involve keto–enolic tautomerization. This pathway is known in the literature and was directly observed by Haugg and co-workers using scanning tunnelling microscopy.^28^ In their work, it was demonstrated that most of the substrate is present as a ketone-enol pair. The enol form, indeed, can be stabilized through hydrogen bonding with a ketone, forming a ketone-enol dimer that acts as an intermediate for the hydrogenation, suggesting a lower-barrier pathway for this reaction with respect to a direct reduction of the carbonyl group. Moreover, the enol form (**2**) of aromatic substrates (**1**) is also stabilized via conjugation with the benzene ring.^28^ Directly observing the enol is challenging, so to prove the involvement of the enol intermediate, the reaction was run over levulinic acid, cyclohexanone, and raspberry ketone: substrates in which the carbonyl is not vicinal to an aromatic ring to stabilize the tautomerization (Table 3).

**Table 3:**
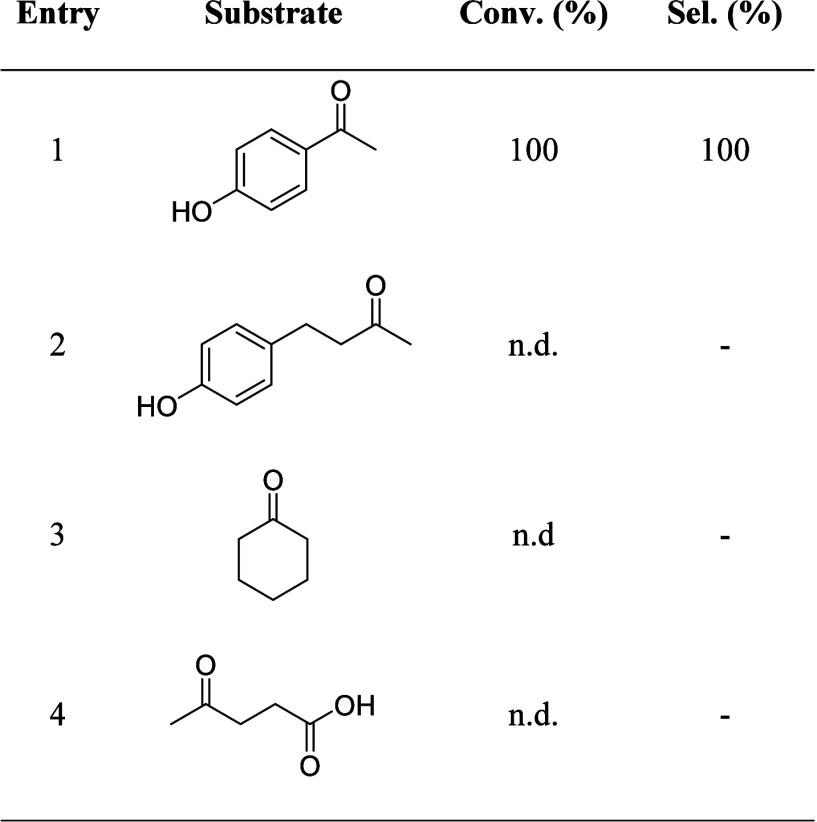
Comparison between the Deoxygenation of 4-HAF and Non-conjugated Ketones^a^

a Reaction conditions: EtOH (1 mL), substrate (2 mmol), catalyst/substrate 1:1000 mol_Pd_/mol, H_2_ (5 bar), 120 °C, 2 h.

**Scheme 2:**
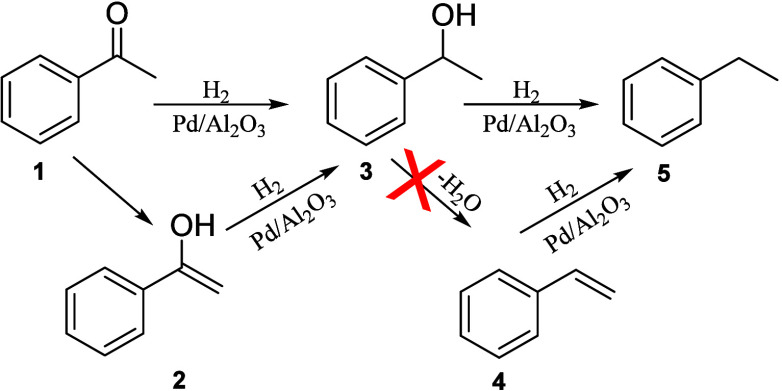
Reaction Mechanism

As expected, no conversion was observed with either substrate. This result suggests that the reduction of enol to alcohol (**3**) is the initial step in the entire reaction. The second step might involve the direct hydrogenation of the alcohol via hydrogenolysis or dehydration of the alcohol over the acidic Al_2_O_3_ support to form an alkene (**4**) first. The second mechanism has been reported for alumina materials.^22,29^ To investigate this possibility, the reaction was run with phenylethanol (**3**) as a model intermediate, with and without H_2_ over either Pd/Al_2_O_3_ or bare alumina. In the absence of H_2_, dehydration would lead to styrene (**4**), which is sufficiently stable to be observed. However, no conversion was detected in both cases. Conversely, complete conversion of phenylethanol to ethylbenzene (**5**) was observed under H_2_, when using Pd/Al_2_O_3_. This indicates that the second step proceeds via direct hydrogenolysis of the alcohol. Indeed, Pd/Al_2_O_3_ was already described to be active in the hydrogenolysis of the C–OH bond.^30^

### Substrate Scope

The reaction was then repeated using EtOH as the solvent over different substrates to expand the substrate scope (Table 4).

**Table 4:**
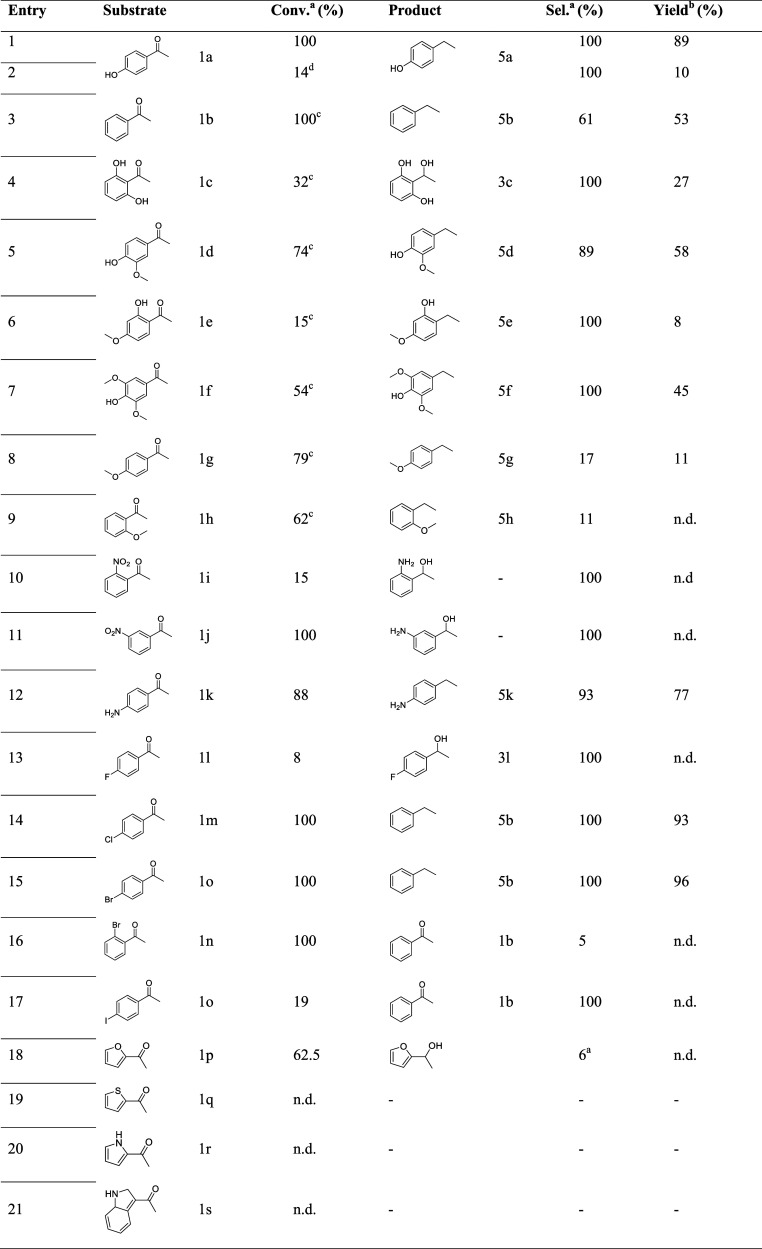
Substrate Scope for the Deoxygenation of Substituted Aromatic Ketones^e^

a GC-MS integrated areas %.

b Isolated yields.

c 4 h.

d Conventional heating.

e Reaction conditions: EtOH (1 mL), substrate (2 mmol), catalyst/substrate 1:1000 mol_Pd_/mol, H_2_ (5 bar), 120 °C, 2 h.

The results in Table 4 highlight a strong correlation between the starting structure and the reaction outcome. The hydrogenation of 4-HAP (**1a**) afforded almost quantitative yields of 4-(1-hydroxyethyl) phenol (**5a**) after 2 h (Table 4, entry 1). However, this reaction was much slower under conventional heating, as a conversion of only 14% was achieved after the same reaction time (Table 4, entry 2). The selectivity toward ethylbenezene (**5b**) was low after 2 h (13%), with phenylethanol being the main product. This result was in accordance with the literature^31^ due to the high energy barrier that hinders the conversion of the hydroxy-alkyl intermediate. Indeed, by doubling the reaction time to 4 h, the selectivity increased to 61% (Table 4, entry 3). However, this time increasing was not so effective in promoting the conversion of 2,6-dihydroxyacetophenone (**1c**), as only 27% of the phenyl ethanol derivative (**3c**, Figure S17) was obtained (Table 4, entry 4). The presence of a methoxy group hinders the reaction (Table 4, entries 5–9), despite this effect not being so detrimental if the −OH phenolic group is still present in the *p*-position (Table 4, entries 5 and 7). This could be explained by phenolic −OH dissociation on the acid–base pairs of Al_2_O_3_ leading to the formation of strongly adsorbed phenolates species at Lewis-acidic Al^3+^ sites,^32^ suggesting that the phenolic species dwelling at/near the perimeter between the metal and the support have an increased chance to react with H species formed on Pd. This strong interaction with the support is sterically hindered in 1-(2-hydroxy-4-methoxyphenyl)­ethan-1-one (**1e**). Despite Pd/Al_2_O_3_ already being described to promote the hydrogenolysis of the C–OH bond,^30^ neither demethoxylation nor hydrodeoxygenation on the aromatic groups was detected.

The presence of the nitro group at the 2- and 3-positions also prevented the reaction (Table 4, entries 10 and 11), leading to the formation of the corresponding amino alcohols (Figures S23 and S24). The subsequent hydrogenolysis reaction of these products was inhibited, as demonstrated by the fact that even when the reaction was prolonged to 4 h, no further conversion of the corresponding amino alcohol was observed.

However, the presence of the amino group at the 4-position boosted the reaction, as the desired product was obtained in a very good yield (Table 4, entry 12). Finally, the effect of halogen groups as substituents was investigated (Table 4, entries 13–17). Dehalogenation was observed in all of the substrates, except for 4-fluoroacetophenone (**1l**), which was converted to a minimal extent into the corresponding alcohol (Figure S26). The conversion was complete only for bromine- and chlorine-substituted acetophenones, affording ethylbenzene as the only product (Table 4, entries 14 and 15). However, a strong steric effect blocked the activity of the catalyst in the hydrogenation of 2-bromoacetophenone (Table 4, entry 16), acetophenone being the main product (Figure S29), as for 4-iodoacetophenone (Table 4, entry 17).

Regarding heterocyclic ketones, the catalytic activity is somewhat challenged. Using acetylfuran (Table 4, entry 18) allowed a satisfactory conversion of 62.5%. However, it is mostly oriented toward the reduction of the aromatic ring yielding the hydrogenated ketone as the primary product. Only a small fraction of 1-(tetrahydrofuran-2-yl)­ethan-1-ol (6%) was observed as a result of carbonyl reduction. For all other heterocyclic substrates (Table 4, entries 19 to 21), no conversion was observed.

## Conclusions

In conclusion, a new MW-assisted process for the synthesis of substituted ethylbenzenes from aromatic ketones has been reported under mild conditions (120 °C, 2 h, 5 bar of H_2_) using commercial Pd/Al_2_O_3_. Experimental insights have been provided to clarify the reaction mechanism, which involves keto–enol tautomerisation followed by direct hydrogenolysis of alcohol to the alkyl chain. The screening of the substrates highlights how polar groups in the *para* position allow high yields (up to 100%) through an improved interaction between the Al_2_O_3_ surface.

## Materials and Methods

All of the starting reagents and catalysts used in this work were purchased from Merck (Darmstadt, Germany).

### Catalyst Characterization

ICP-OES analysis on the commercial Pd/Al_2_O_3_ catalyst, as well as after the first and the fifth reaction cycles, was performed with a PerkinElmer Optima 7000 (PerkinElmer, Norwalk, CT, USA) spectrometer.

The powder X-ray diffraction (PXRD) pattern was acquired by using a PW3050/60 X’Pert PRO MPD diffractometer (PANalytical) working in Bragg–Brentano geometry, using as a source the high-powered ceramic tube PW3373/10 LFF with a Cu anode (employing Cu Kα1 radiation λ = 1.5406 Å) equipped with a Ni filter to attenuate Kβ. Scattered photons were collected by a real time multiple strip (RTMS) X’celerator detector. Data was collected in the 10° ≤ 2θ ≤ 90° angular range, with 0.02° 2θ steps. The powdered sample was examined in its as-received form and placed in a spinning sample holder in order to minimize preferred orientations of crystallites.

Field emission scanning electron microscopy (FESEM) measurements were performed by using a TESCAN S9000G FESEM 3010 microscope (30 kV), equipped with a high brightness Schottky emitter and energy-dispersive X-ray spectroscopy (EDS) analysis thanks to an Ultim Max Silicon Drift Detector (SDD, Oxford, Abingdon-on-Thames, UK). The sample was deposited on a stub that was coated with a conducting adhesive and inserted into the chamber by a fully motorized procedure. Before the measurements, the sample was submitted to metallization with Cr (ca. 5 nm) to avoid any charging effect due to the possible presence of reaction products and intermediates (Emitech K575X sputter coater). The histogram of the Pd particle size distribution was obtained by considering a representative number of particles and the mean particle diameter (*d*_m_) was calculated by applying the following equation:

*d*_m_ = Σ*d_i_n_i_*/Σ*n_i_*, with *n_i_* being the number of particles with diameter *d_i_*.

### Deoxygenation Reactions

For the microwave-assisted synthesis, a SynthWAVE (Milestone srl, Ceresole, Italy) sealed reactor was used. SyntWAVE provides dielectric heating up to 250 °C and pressure up to 300 bar. The reaction mixtures were prepared in 15 mL quartz vials, placed in a PTFE reaction chamber of 1 L in volume. A brine solution (200 mL) was added to ensure optimal absorption of the microwave radiation. The temperature control is performed with a thermocouple directly immersed in the brine solution. Mixing is provided via magnetic stirring up to 500 rpm. The heating ramp was set to 2 min for each test. The instrument is set to work with 1.5 kW of maximum power at 2.5 GHz and the software automatically manages the power output to follow the programmed temperature.

As a general procedure, the starting ketone (2 mmol) was inserted into a 15 mL quartz vial equipped with a 15 mm magnetic stirring bar vial and a commercial Pd/Al_2_O_3_ (PN: 1003371705; 5 wt %) catalyst (1:1000 Pd to substrate molar ratio) was inserted. The solvent of choice (1 mL) was slowly added, and a PTFE cap was placed on the vial. The caps used present a small hole on the side to allow homogenization with the external reactive atmosphere. Before the reaction, the reactor was purged with dry N_2_, then H_2_ (5 bar) was introduced at room temperature, and the chamber was sealed for the experiment.

After the reaction, the crude product was transferred to test tubes for centrifugation at 4200 rpm for 2 min. The supernatant was collected and diluted in CHCl_3_ for GC-MS analysis.

For the experiment under conventional heating, equipment for pressurized gas was provided by Labtech SRL (Sorisole, Italy). The equipment consists of a 10 mL Teflon reaction chamber, an aluminum jacket to provide mechanical stability and heat transfer, a sealing cap provided with a manometer for pressure control up to 15 bar, and a valve for introducing and releasing the pressurized gases. Heating and stirring were provided by a Xelsius reactor (LabTech SRL). This reactor allows heating from −20 to 150 °C and is endowed with magnetic stirring (800 rpm).

### Flash Chromatography

To obtain the isolated product, the crude was dissolved in CHCl_3_ with the addition of C18 silica (LiChroprep RP18) to obtain a dry powder, which was packed in a sealed precolumn. The flash chromatograpy system is a PuriFlash 5.050 (Advion Interchim Scientific, USA), and the column used for the separation is a 4 g C18 catridge Purezza Daily (SepaChrom, Italy). The peaks were detected at 254 and 280 nm and automatically collected. Water (A) and methanol (B) were used as mobile phases as follows (Table 5):

**Table 5:** Gradient for the Purification of Reaction Crudes by Flash Chromatography

**CV**	**A (%)**	**B (%)**
0	80	20
2	80	20
12	20	80
16	20	80
18	0	100
20	0	100

### GC-MS Analysis

The identification of the reaction products was performed via GC-MS analysis. The system used is an Agilent Technologies 6850 Network GC System fitted with a 5973 Network Mass Selective Detector, a 683B Automatic Sampler, and a capillary column Mega 5MS (length 30 m; i.d. 0.25 mm; film thickness 0.25 μm, Mega s.r.l., Legnano, Italy).

Typically, 1–2 mg/mL samples were prepared via dilution in CHCl_3_ for analysis. For some products with free oxidryl groups, derivatization with bis-trimethysilylacetamide (BSA) was necessary. For the derivatization, a 2:1 molar excess of BSA was added to the diluted crude, and the solution was left to react at 60 °C for 1 h.

### NMR Analysis

NMR analyses were performed with a 600 MHz JEOL JNM-ECZ600R (JEOL, Tokyo, Japan) spectrometer. The dried sample (about 20 mg) was dissolved in CDCl_3_ (700 μL) and analyzed. NMR spectra were carried out only for products isolated with yields >5%.

## Supplementary Material



## Notes

Experimental procedures, analytical and spectroscopic data, and copies of NMR spectra (PDF)

## †

Departamento de Química Inorgánica, Instituto de Investigaciones Quıímicas and Centro de Innovación en Química Avanzada (ORFEO–CINQA), Centro mixto CSIC-Universidad de Sevilla, 41092 Sevilla, Spain
