# Supplementary material for: Microwave-Assisted Deoxygenation of Substituted Aromatic Ketones over Commercial Pd/Al2O3 under Mild Conditions
Source: ACS Omega. 2026 Mar 12;11(11):17874–80. doi: 10.1021/acsomega.5c12401 (PMC13019252; doi:10.1021/acsomega.5c12401)
Supplement: Supplementary file 1 [file ao5c12401_si_001.pdf]

MW-assisted deoxygenation of substituted aromatic ketones over commercial Pd/Al_2O_3 under mild conditions

Fabio Bucciol, Ignacio C. Vega, Emanuela C. Gaudino, Maela Manzoli, Silvia Tabasso,* Giancarlo Cravotto

University of Turin, Department of Drug Science and Technology, Via Pietro Giuria 9, 10125, Turin.

Characterization of the commercial Pd/Al_2O_3 catalyst

The PXRD pattern of the commercial Pd/Al_2O_3 catalyst is shown in Figure S1, panel a. Peaks related to the alumina support mainly in the cubic phase (00-002-1421), but also in the hexagonal (00-002-0921) and rhombohedral (00-002-1227) phases are observed. No peaks due to crystalline Pd have been detected, likely indicating high metal dispersion. This is confirmed by looking at the FESEM images (Figure S1, panels b and c) which revealed the presence of Pd nanoparticle agglomerates covering the support (panel b) accompanied by highly dispersed Pd nanoparticles with mean diameter d_m= 5.6 ± 1.1 nm (panel d).

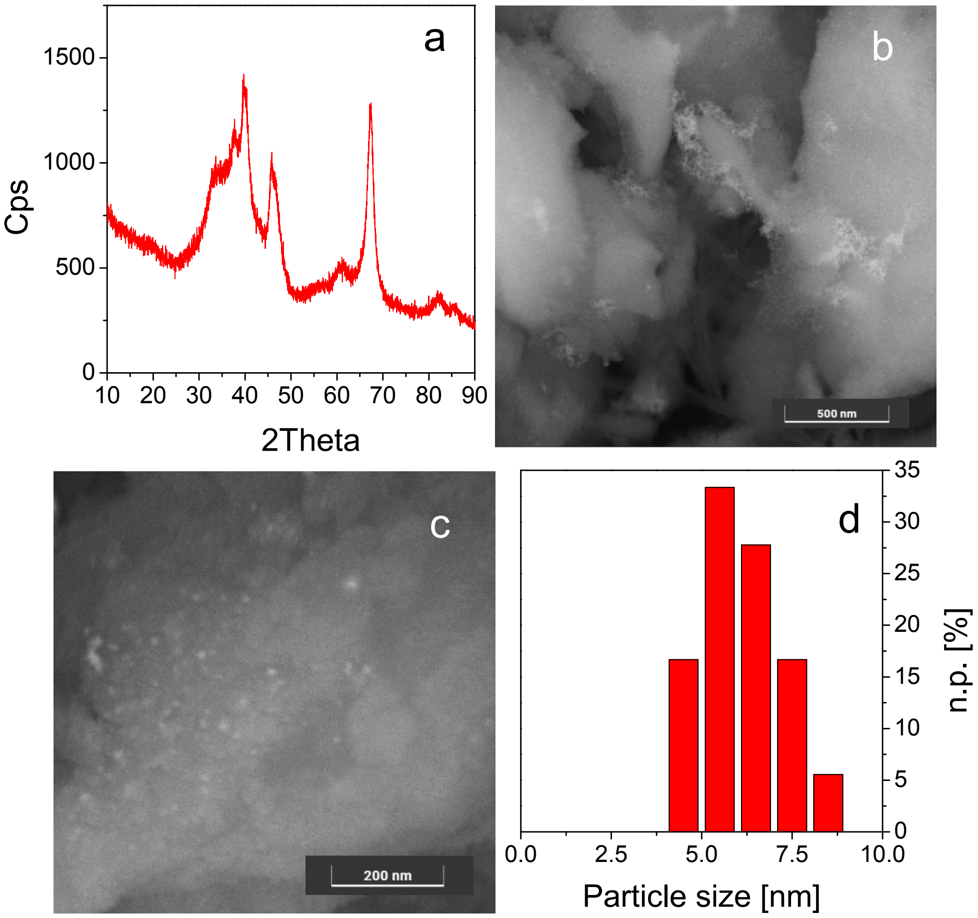

Figure S1: PXRD pattern (a) and FESEM representative images of commercial Pd/Al_2O_3 catalyst acquired in BSE mode in which the Pd particles appear brighter with respect to the alumina support (b, c). Instrumental magnification 133000× and 300000×. Pd particle size distribution (d), n.p. [%] represents the number of counted particles of diameter d_i.

Products characterization

4-ethylphenol (5a). White solid (217 mg, 89% yield). GC-MS identification: retention time: 13.150 min. mw: 122. Observed *m/z* 294 (M+ trimethylsylil), 179, 151, 135, 121, 105, 91, 73, 59, 45, 27, 15.

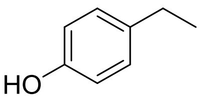

^{1}H NMR (600 MHz, $CDCl_3$): δ 7.06 (d, J = 8.6 Hz, 2H), 6.75 (d, J = 8.6 Hz, 2H), 4.98 (br s, 1H), 2.57 (q, J = 7.6 Hz, 2H), 1.19 (t, J = 7.6 Hz, 3H). ^{13}C NMR (151 MHz, $CDCl_3$): δ 153.5, 136.6, 129.0, 115.2, 28.0, 15.9. These data are in agreement with literature.[1]

Ethylbenzene (5b). Colorless oil (112 mg, 53% yield). GC-MS identification: retention time: 6.210 min. MW: 106. Observed m/z: 106, 91, 77, 65, 51, 39, 27, 15.min.

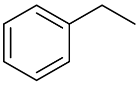

^{1}H NMR (600 MHz, $CDCl_3$): δ 7.34 – 7.14 (m, 5H), 2.64 (q, J = 7.6 Hz, 2H), 1.23 (t, J = 7.6 Hz, 3H). ^{13}C NMR (151 MHz, CDCl3): δ 144.3, 128.3, 127.9, 125.7, 28.9, 15.7. These data are in agreement with literature.[2]

4-ethyl-2-methoxyphenol (5d). Colorless oil (176 mg, 58%). GC-MS identification: retention time: 14.150 min. MW: 152. Observed m/z: 152, 137, 109. 91, 75, 51, 15.

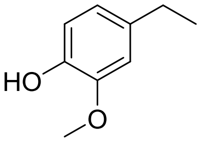

^{1}H NMR (600 MHz, $CDCl_3$): δ 6.84 (d, J = 7.9 Hz, 1H), 6.70 (d, J = 8.8 Hz, 2H), 3.88 (s, 3H), 2.59 (q, J = 7.6 Hz, 2H), 1.22 (t, J = 7.6 Hz, 3H). ^{13}C NMR (151 MHz, $CDCl_3$): δ 146.5, 143.6, 136.4, 120.4, 114.3, 110.6, 55.9, 28.6, 16.0. These data are in agreement with literature.[3]

2-ethyl-5-methoxyphenol (5e). Colorless oil (24 mg, 8%). GC-MS identification: Retention time: 15.109 min. MW: 152. Observed m/z: 152, 137, 121, 109, 91, 77, 65, 51, 39, 28, 18.

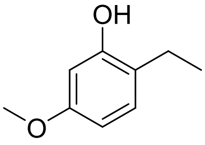

^{1}H NMR (600 MHz, $CDCl_3$): δ 7.03 (d, J = 8.4 Hz, 1H), 6.45 (dd, J = 8.3, 2.6 Hz, 1H), 6.39 (d, J = 2.6 Hz, 1H), 3.74 (s, 3H), 2.58 (q, J = 7.6 Hz, 2H), 1.21 (t, J = 7.6 Hz, 3H). ^{13}C NMR (151 MHz, $CDCl_3$): δ 158.8, 154.4, 129.8, 122.7, 105.9, 101.9, 55.4, 22.4, 14.4.

4-hydroxy-3,5-dimethoxyethylbenzene (5f). Colorless oil (164 mg, 45%). GC-MS identification: Retention time: 16.123 min. MW: 182. Observed m/z: 182, 167, 151, 139, 123, 107, 91, 77, 67, 53, 39, 27, 15.

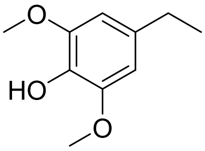

^{1}H NMR (600 MHz, $CDCl_3$): δ 6.41 (s, 2H), 3.87 (s, 6H), 2.57 (q, J = 7.6 Hz, 2H), 1.21 (t, J = 7.6 Hz, 3H). ^{13}C NMR (151 MHz, $CDCl_3$) δ 147.0, 135.5, 132.7, 104.5, 56.3, 29.1, 16.0.

4-methoxyethylbenzene (5g). Colorless oil (29 mg, 11%). GC-MS identification: retention time: 11.447 min. MW: 136. Observed m/z: 136, 121, 105, 91, 77, 65, 51, 39, 28, 18.

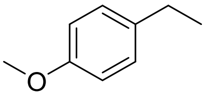

^1H NMR (600 MHz, $CDCl_3$) δ 7.26 (d, J = 8.7 Hz, 2H), 6.84 (d, J = 8.6 Hz, 2H), 3.77 (s, 3H), 1.43 (q, J = 6.6 Hz, 2H), 1.19 (t, J = 6.6 Hz, 3H). ^{13}C NMR (151 MHz, $CDCl_3$): δ 158.9, 138.2, 126.7, 113.9, 55.3, 25.1, 18.4.

4-aminoethylbenzene (5k). Colorless oil (186 mg, 77% yield). GC-MS identification: Retention time 12.386 min. MW: 121. Observed m/z: 121, 106, 91, 77, 65, 58.5, 53, 39, 28, 18.

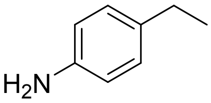

^1H NMR (600 MHz, $CDCl_3$): δ 6.98 (d, J = 8.6 Hz, 1H), 6.62 (d, J = 8.4 Hz, 2H), 2.53 (q, J = 7.6 Hz, 2H), 1.18 (t, J = 7.6 Hz, 4H). ^{13}C NMR (151 MHz, CDCl3) 144.11, 134.58, 128.68, 115.37, 28.06, 16.03.

NMR Spectra

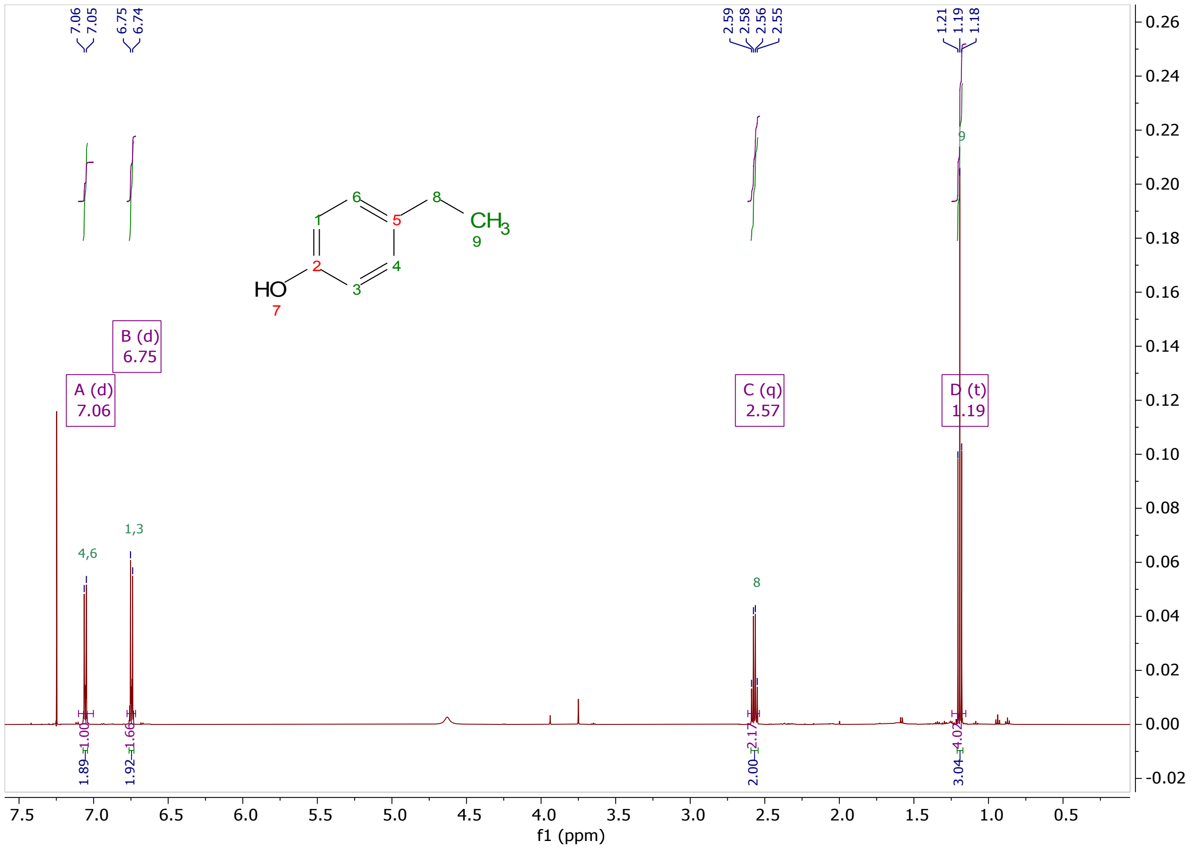

Figure S2: ^{1}H NMR (600 MHz, $CDCl_3$) of **5a**.

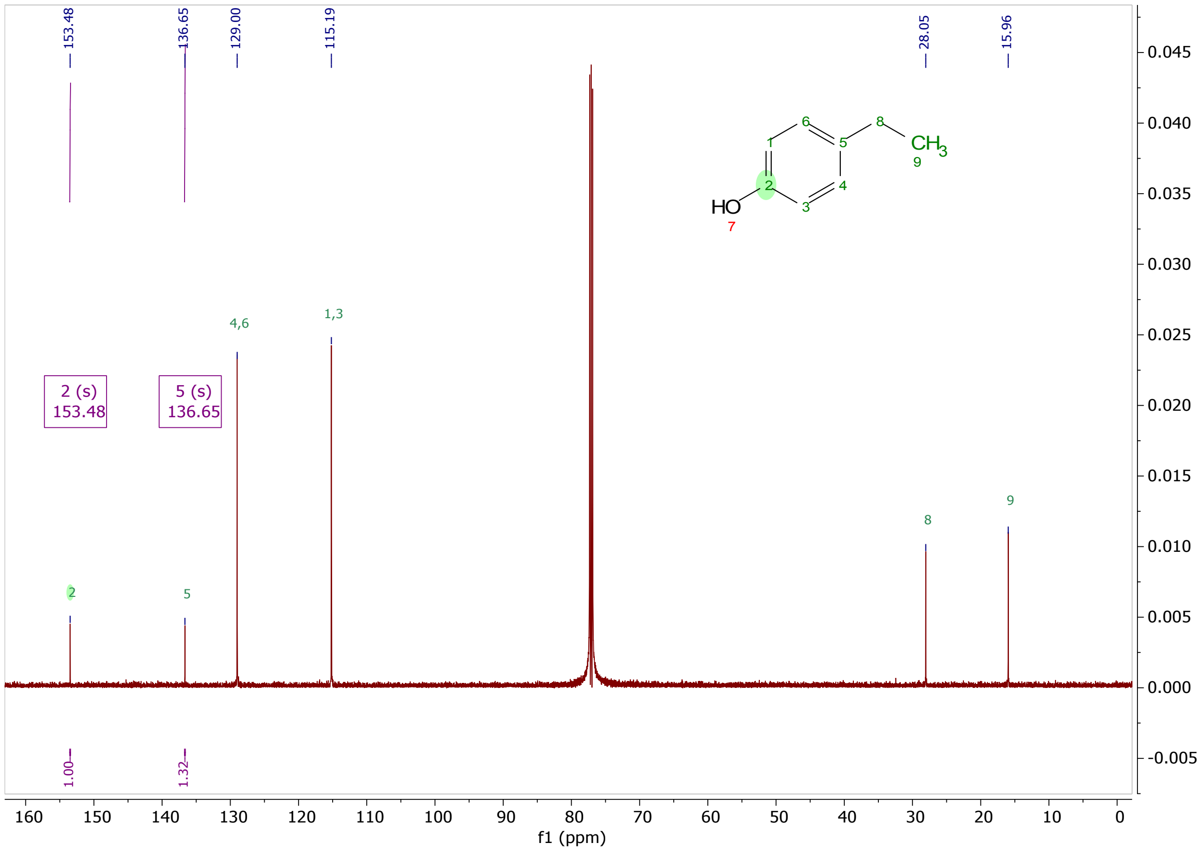

Figure S3: ^{13}C NMR (151 MHz, CDCl3) of **5a**.

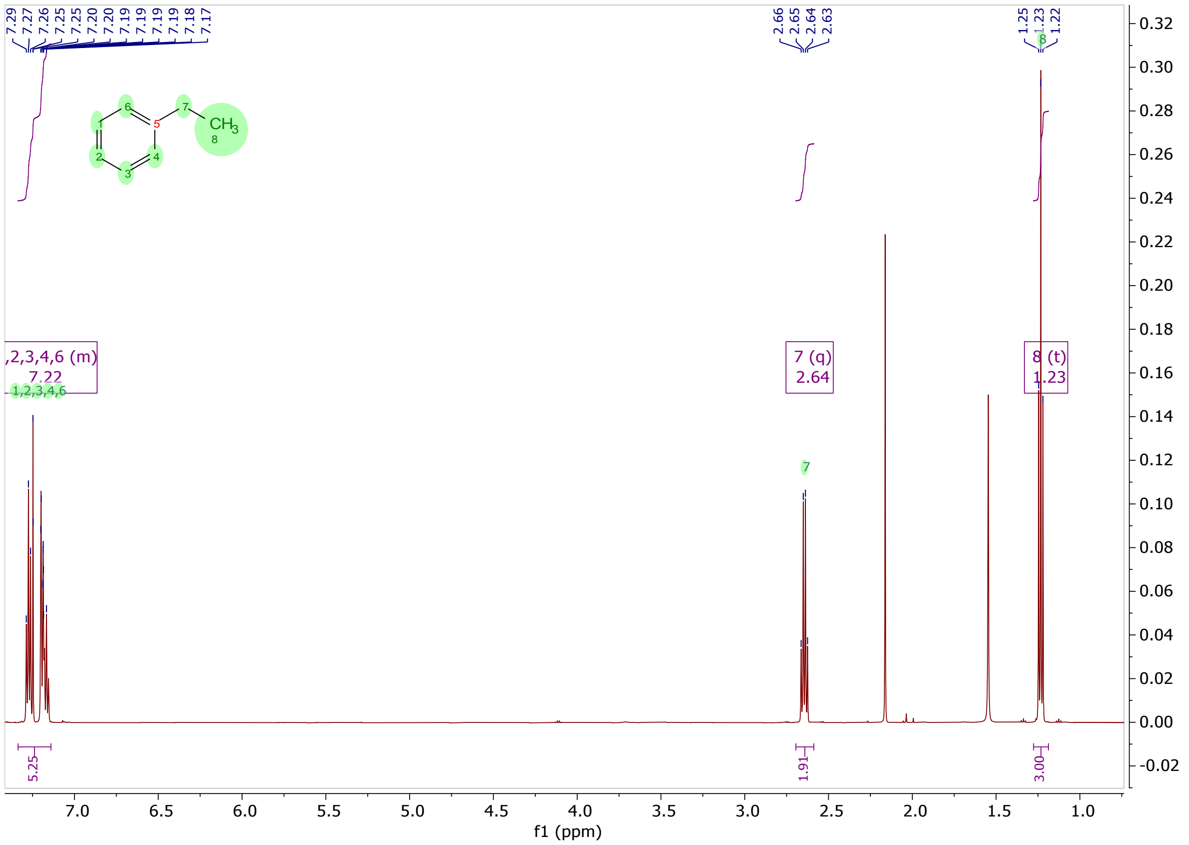

Figure S4: ^{1}H NMR (600 MHz, $CDCl_3$) of **5b**.

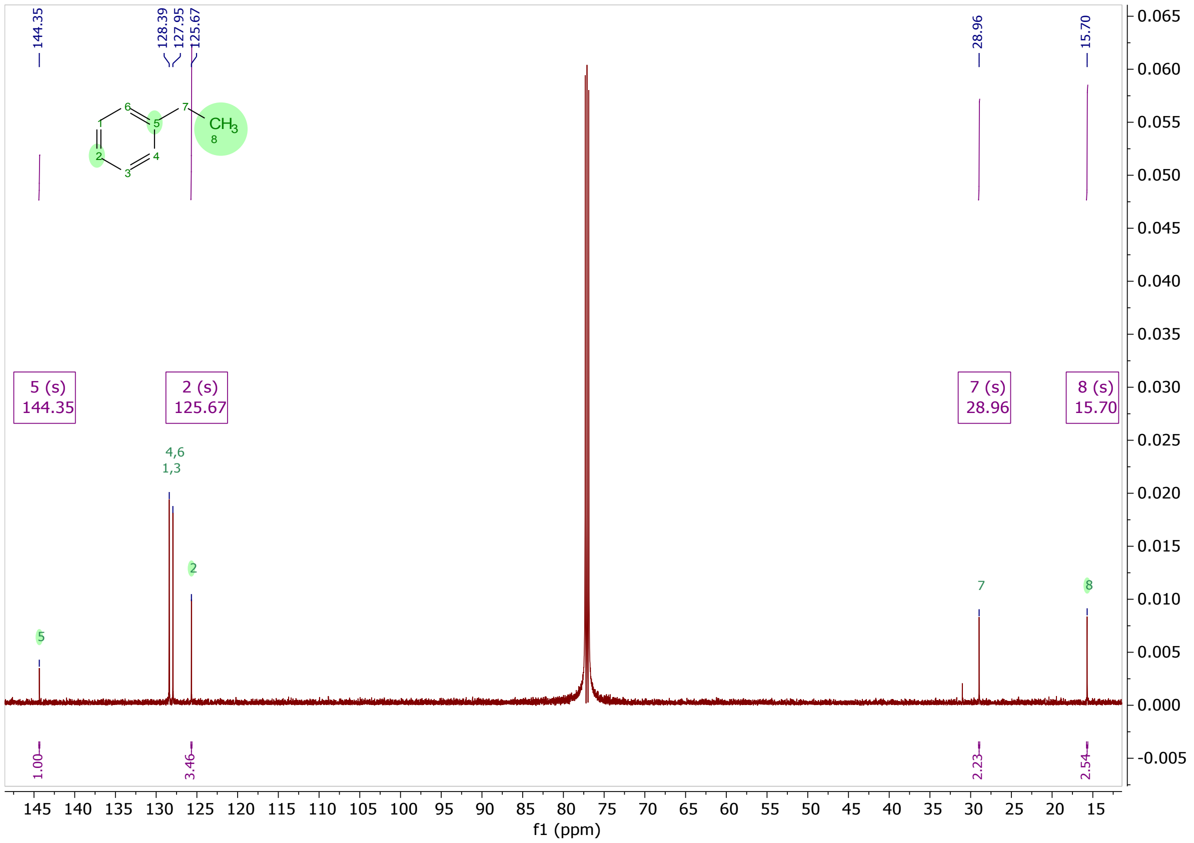

Figure S5: ^{13}C NMR (151 MHz, $CDCl_3$) of **5b**.

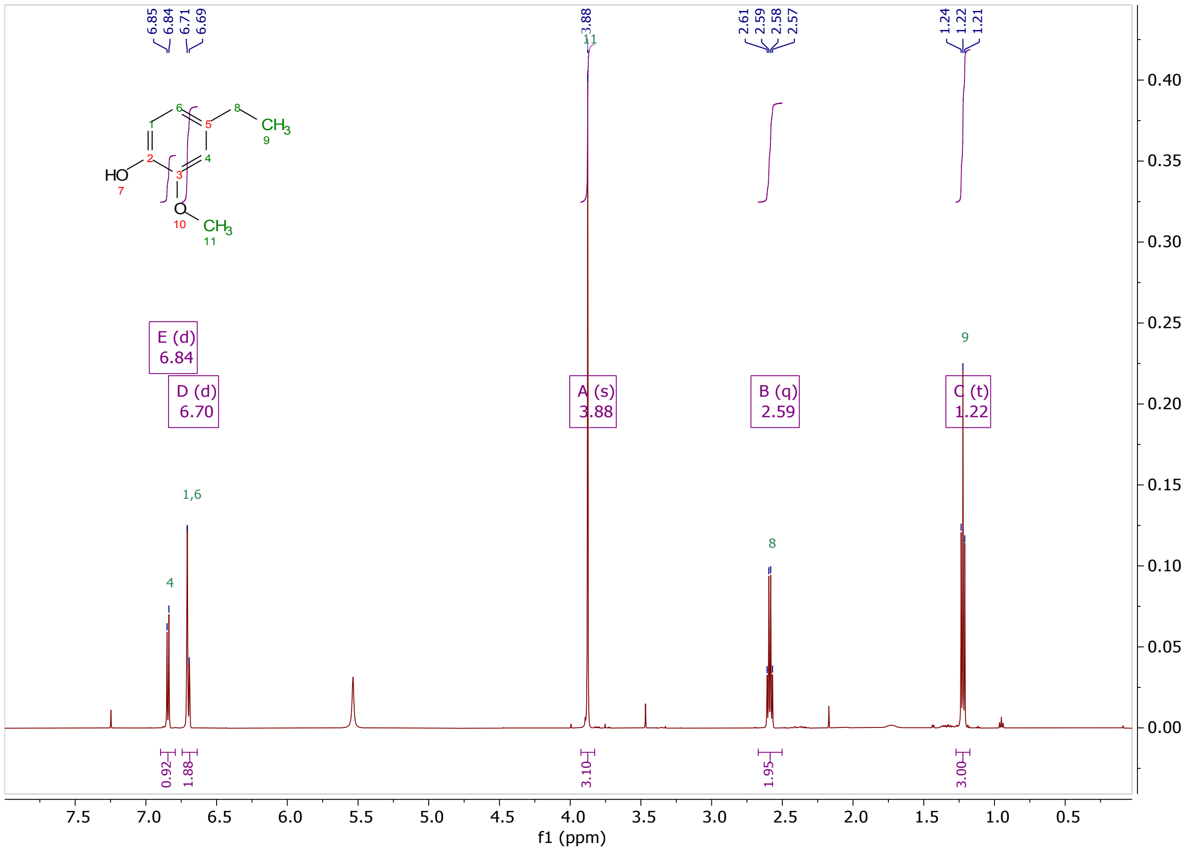

Figure S6: ^{1}H NMR (600 MHz, $CDCl_3$) of **5d**.

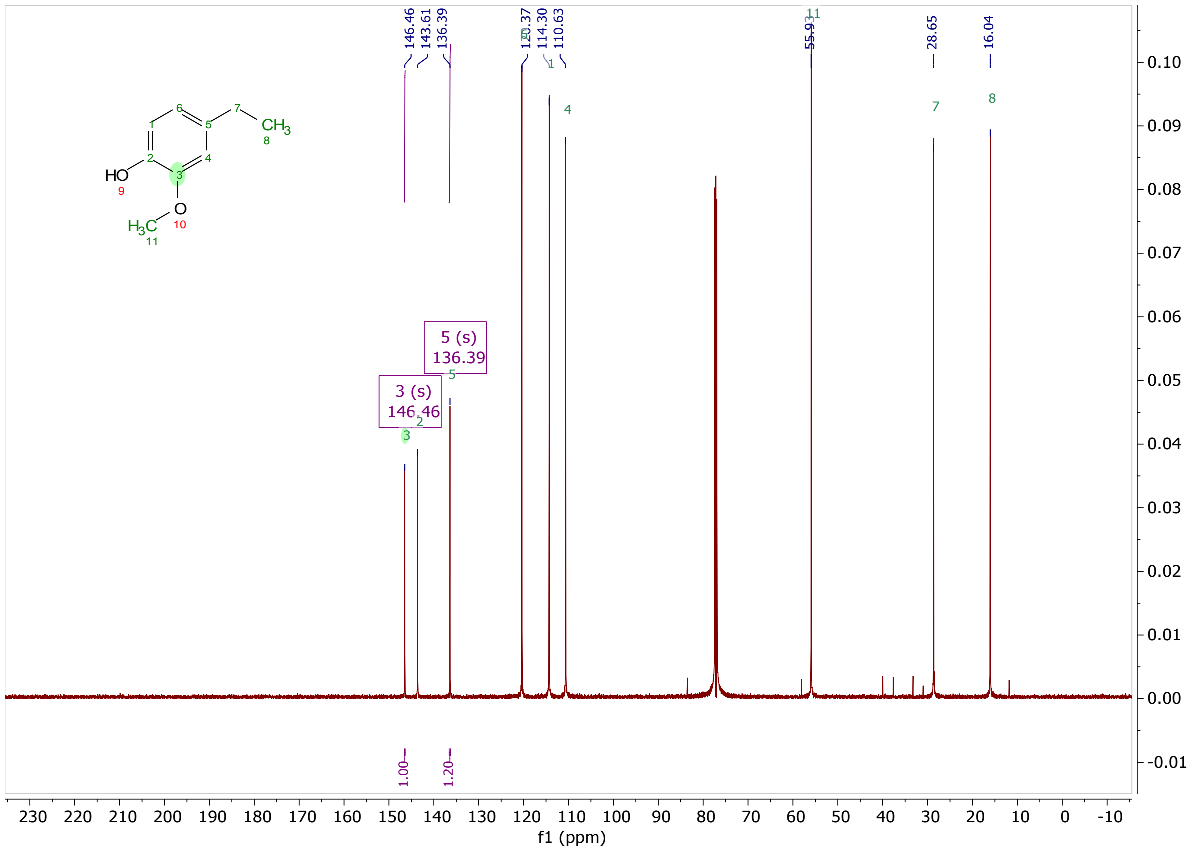

Figure S7: ^{13}C NMR (151 MHz, $CDCl_3$) of **5d**.

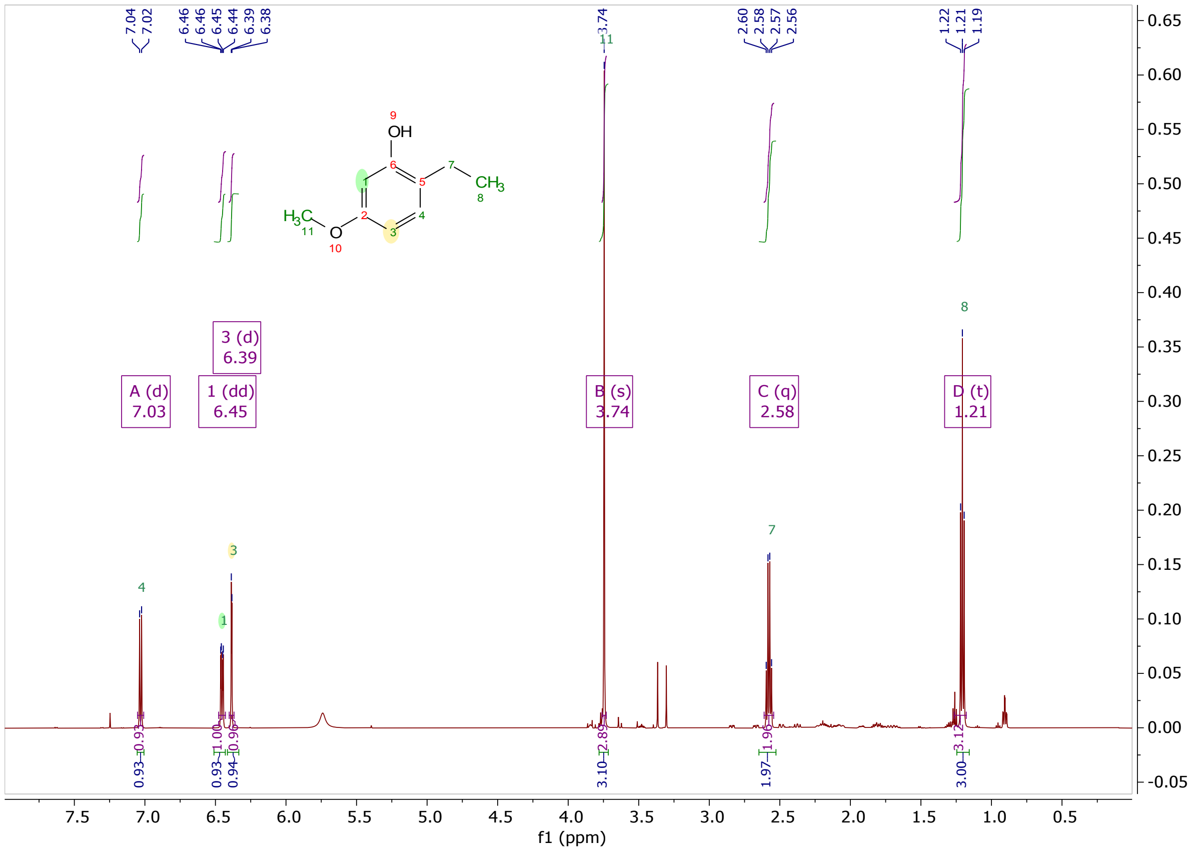

Figure S8: ^{1}H NMR (600 MHz, $CDCl_3$) of **5e**.

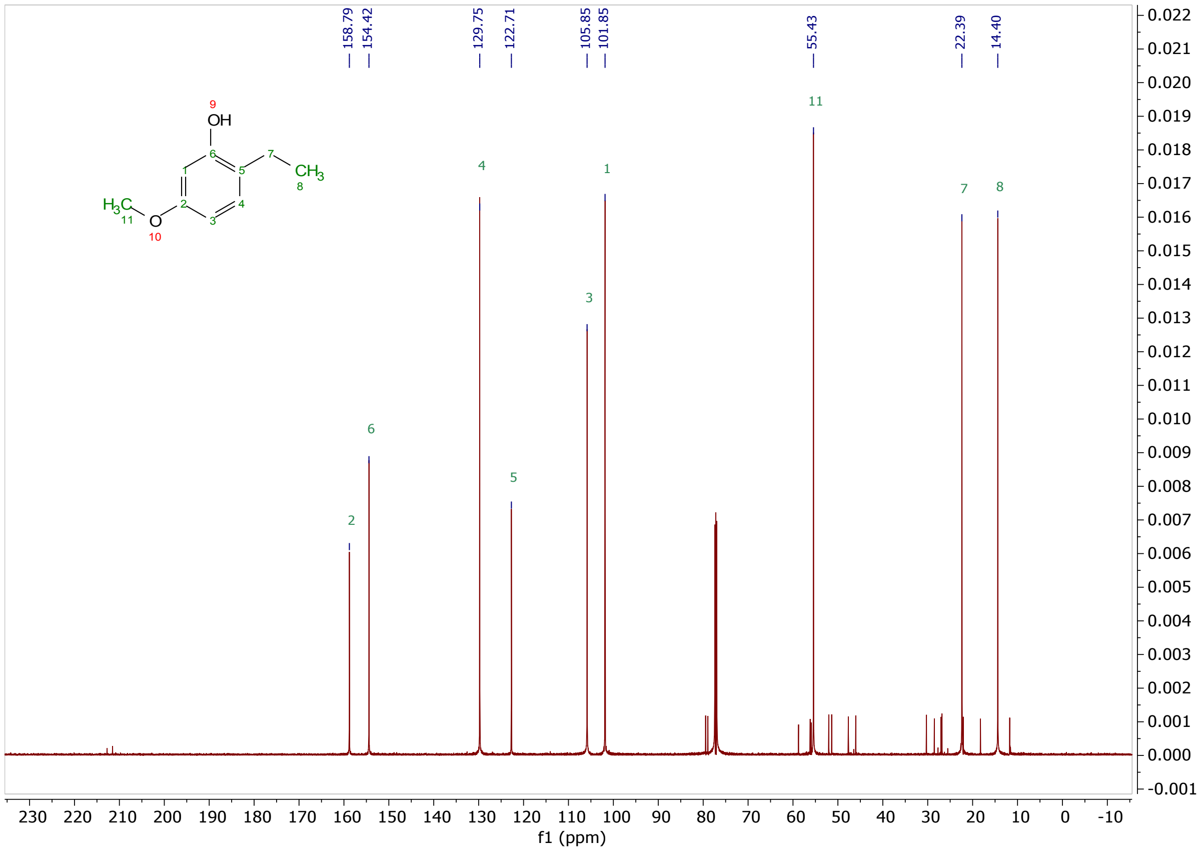

Figure S9: ^{13}C NMR (151 MHz, $CDCl_3$) of **5e**.

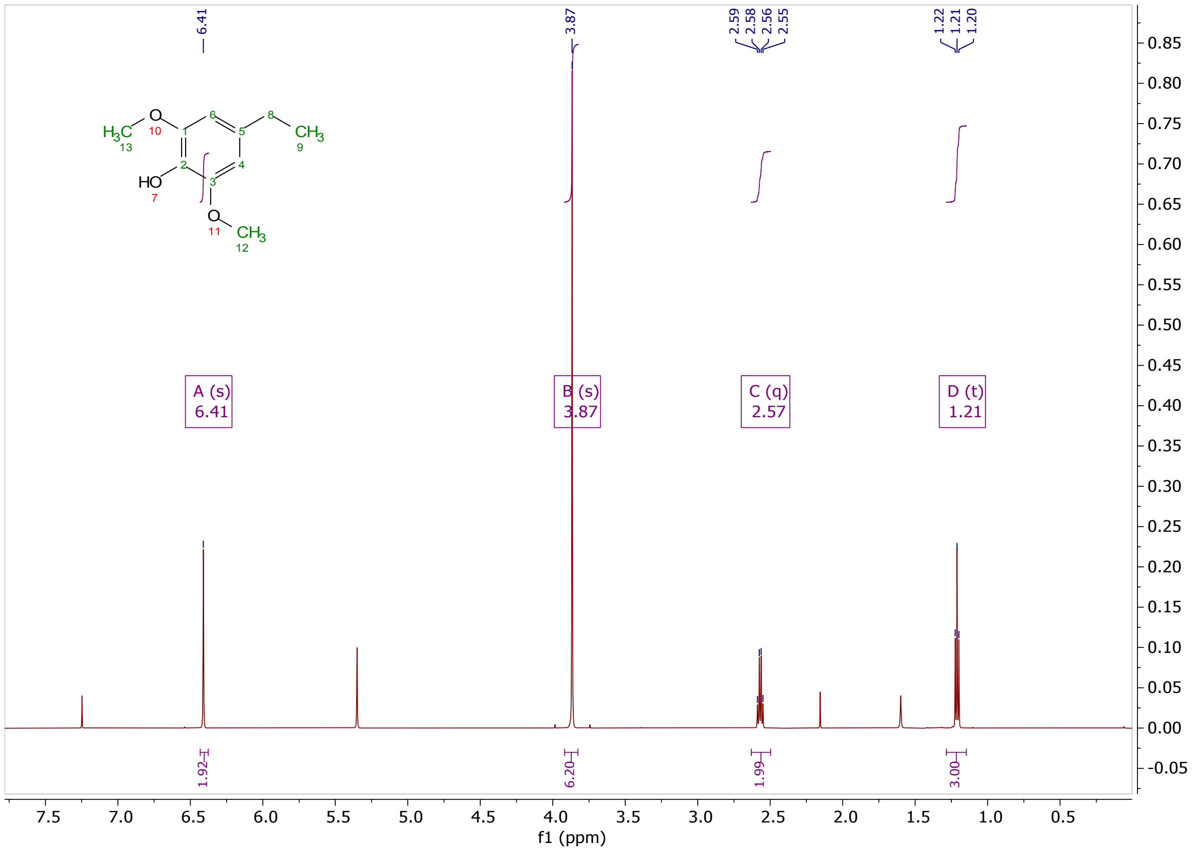

Figure S10: ^{1}H NMR (600 MHz, $CDCl_3$) of **5f**.

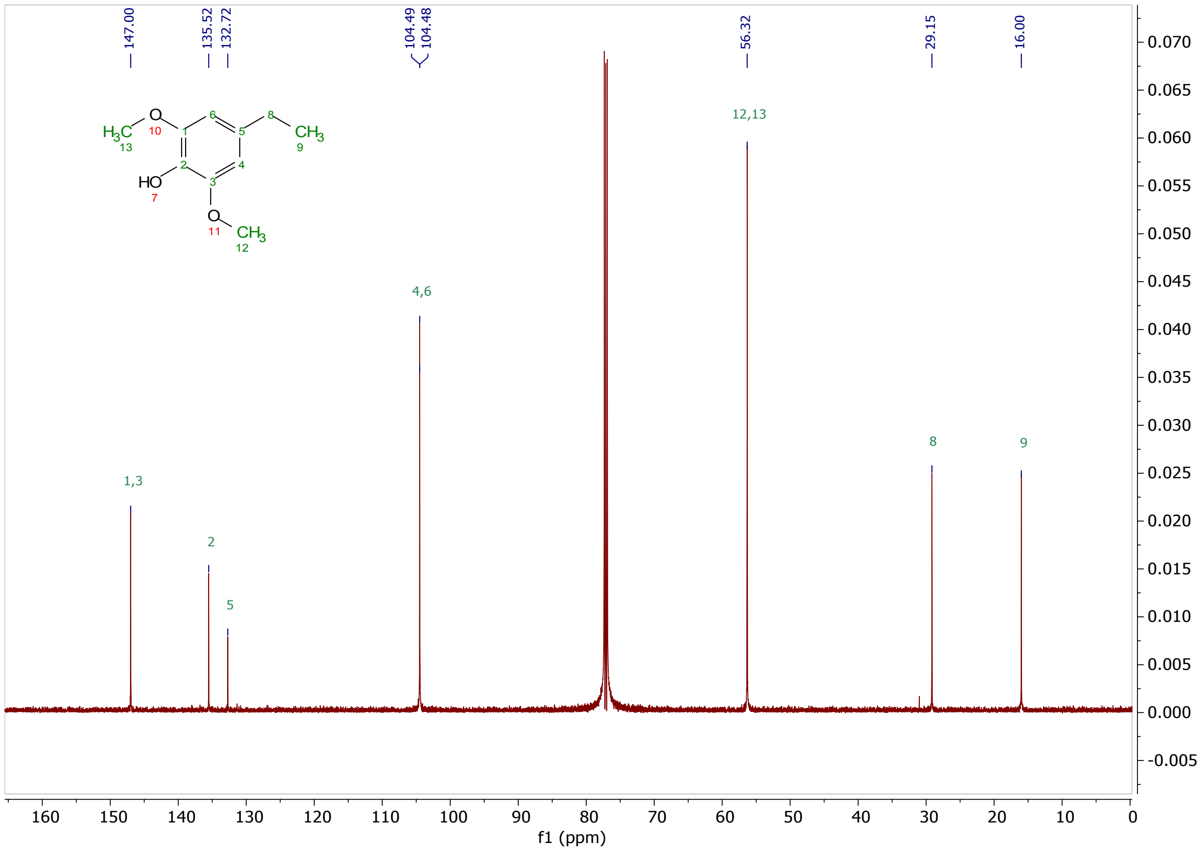

Figure S11: ^{13}C NMR (151 MHz, $CDCl_3$) of **5f**.

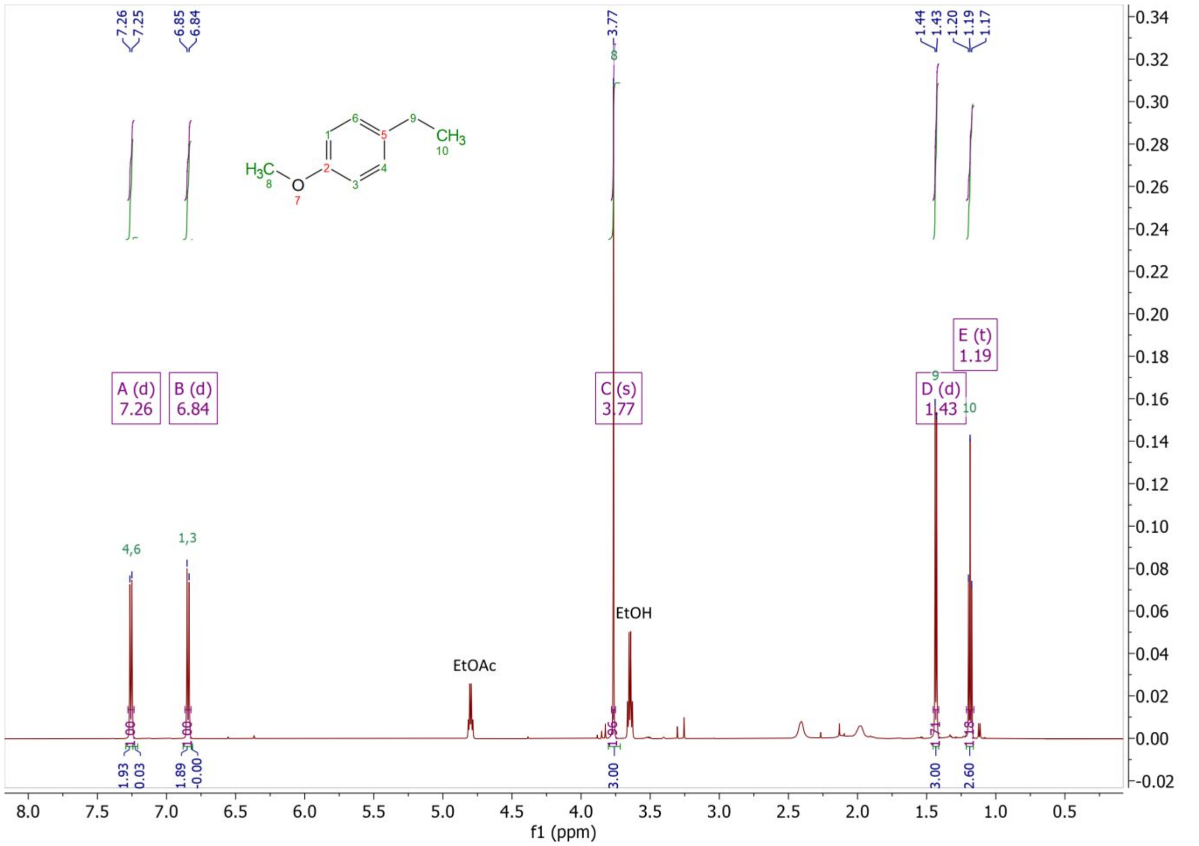

Figure S12: ^{1}H NMR (600 MHz, $CDCl_3$) of **5g**.

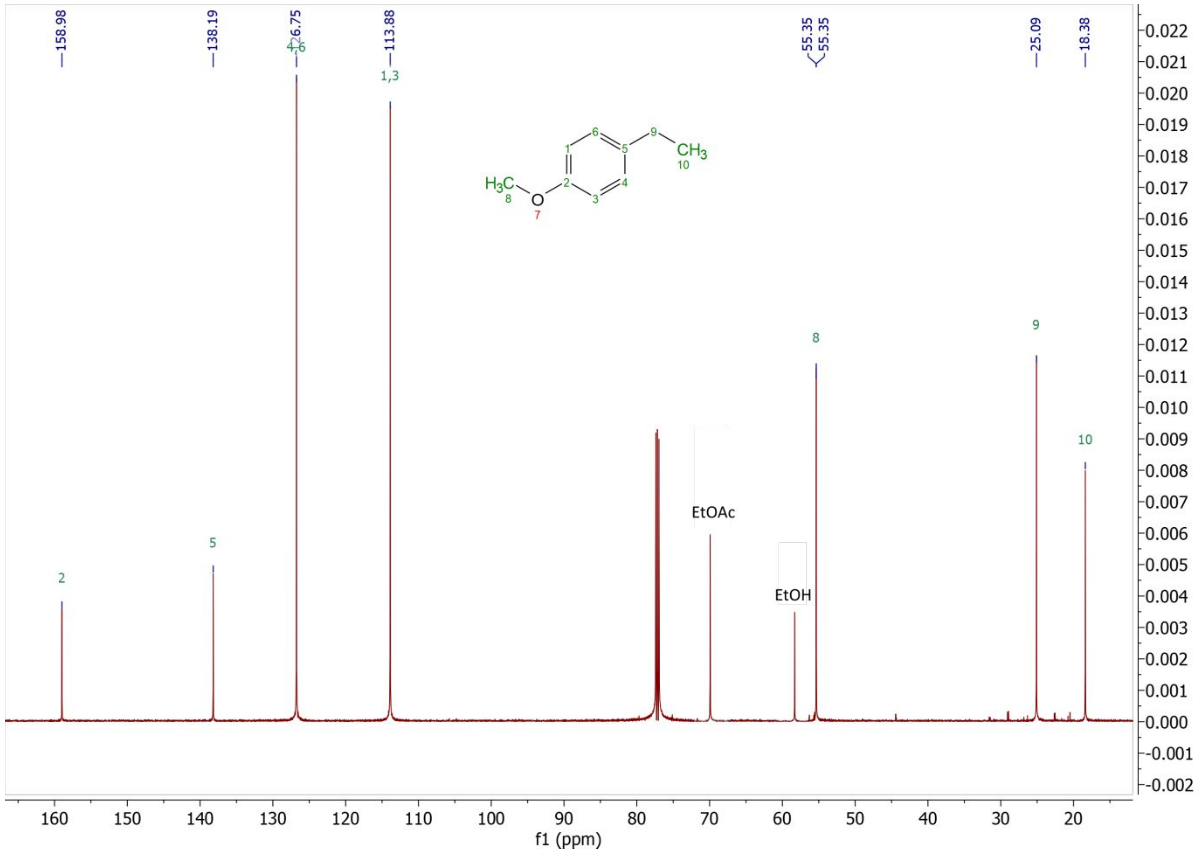

Figure S13: ^{13}C NMR (151 MHz, $CDCl_3$) of **5g**.

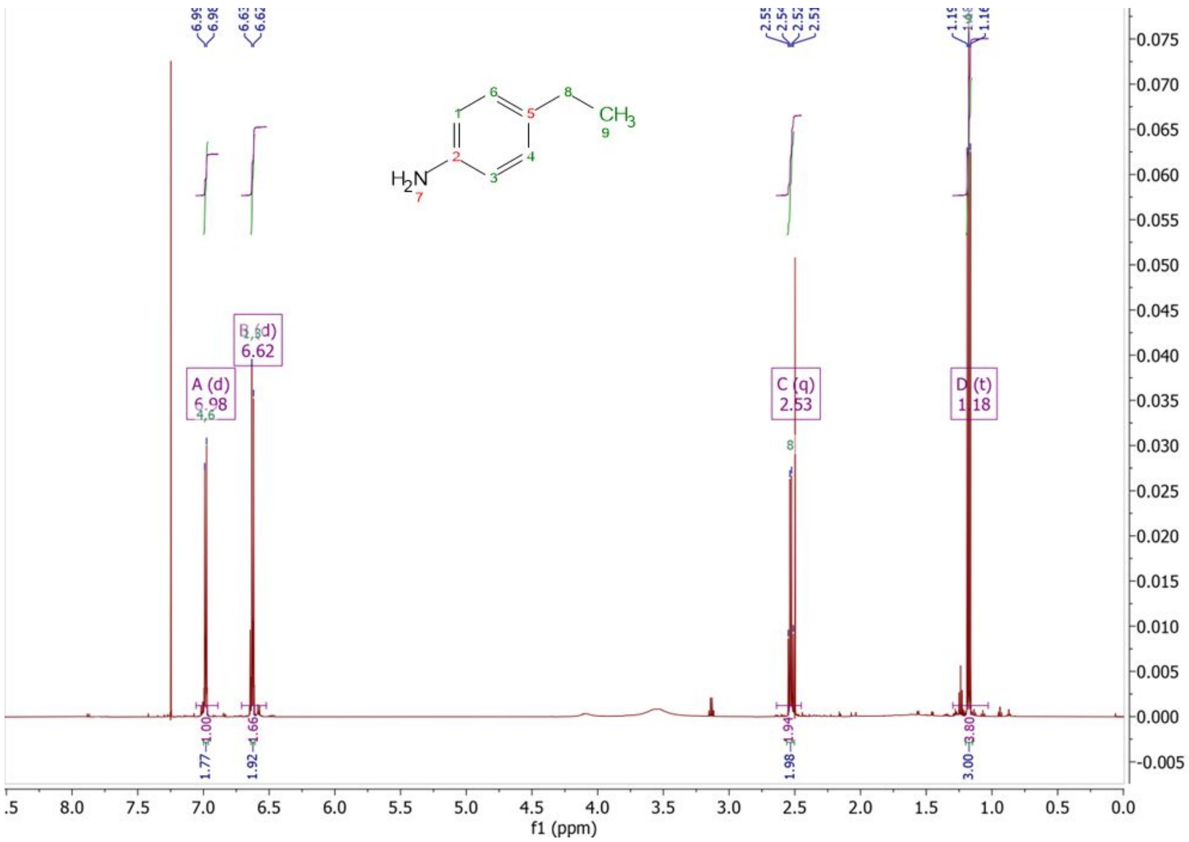

Figure S14: ^{1}H NMR (600 MHz, $CDCl_3$) of 4-aminoethylbenzene (**5k**).

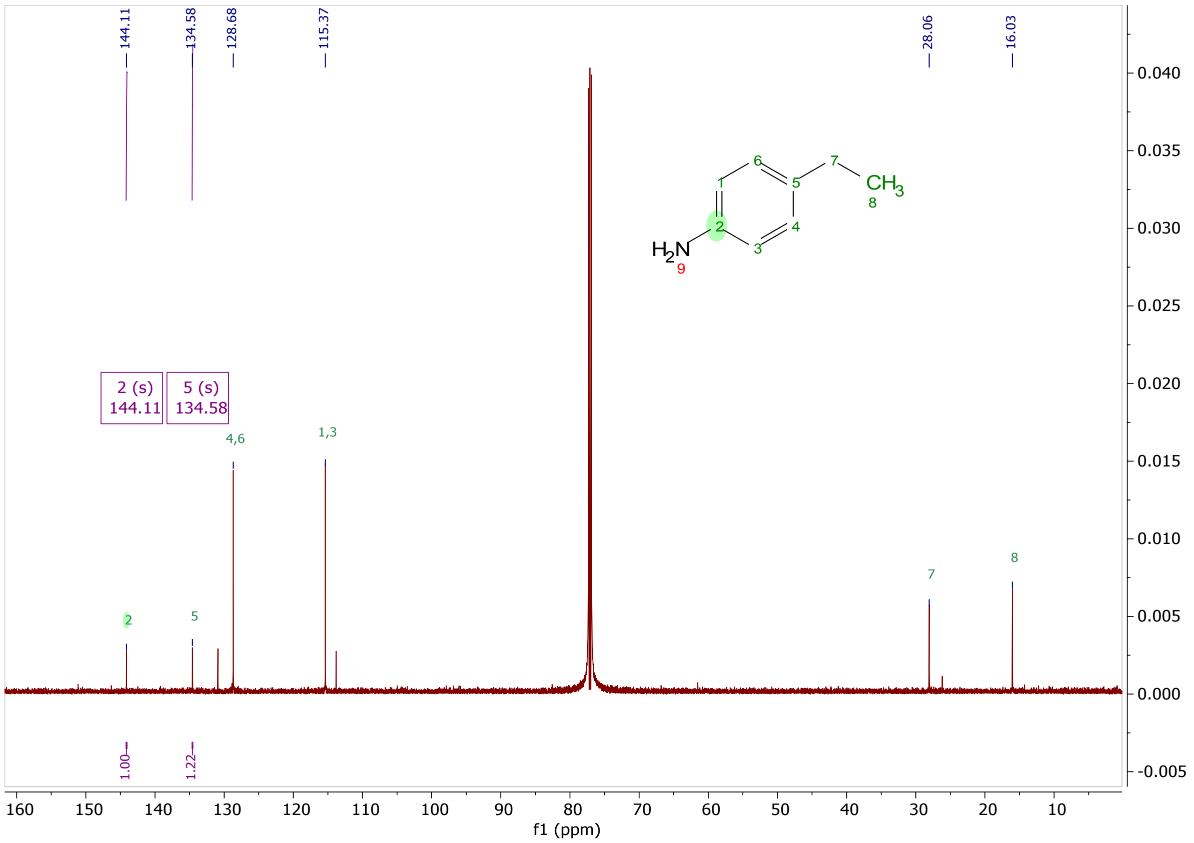

Figure S15: ^{13}C NMR (151 MHz, $CDCl_3$) of 4-aminoethylbenzene (**5k**).

GC-MS Spectra

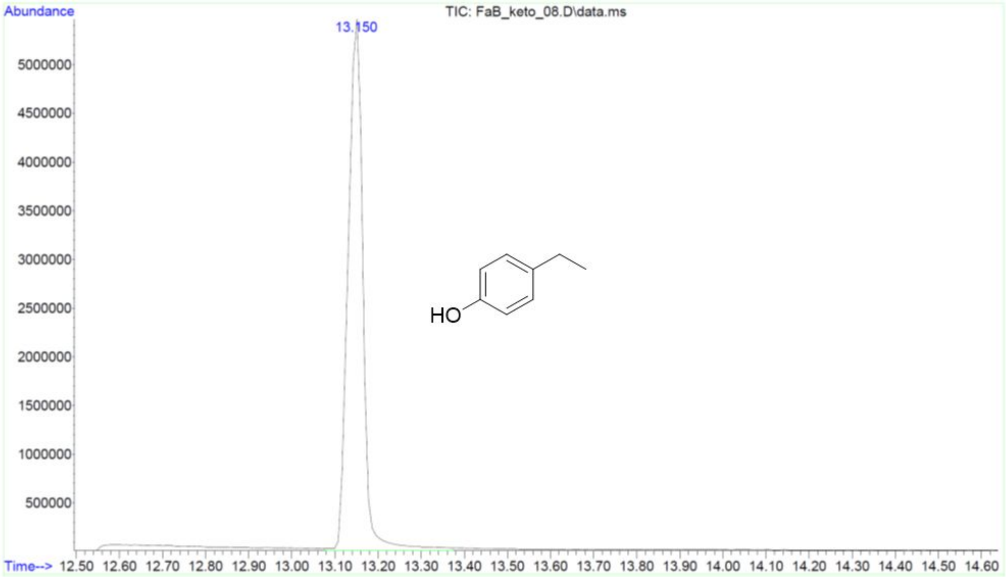

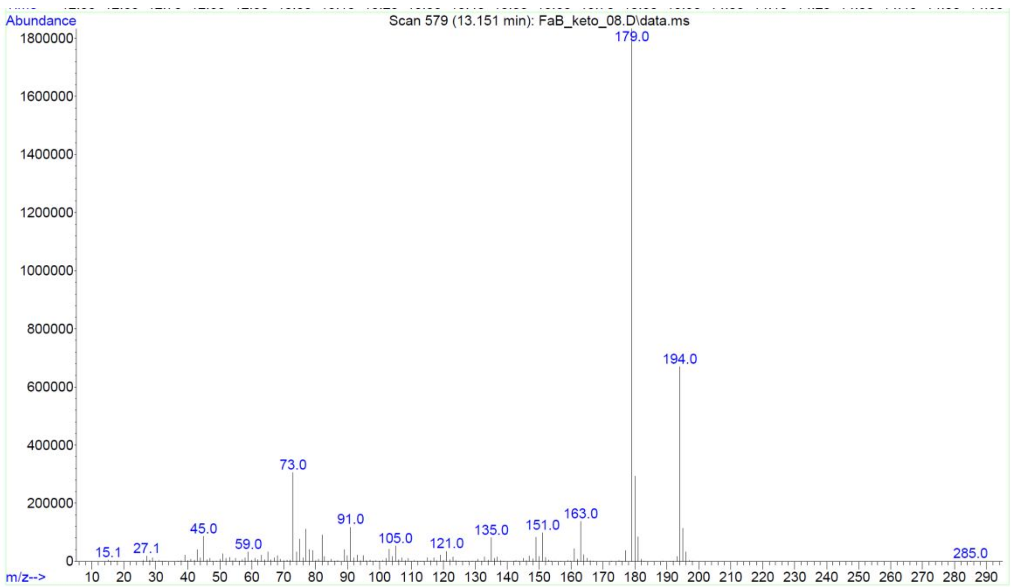

Figure S16: GC-MS identification of 4-hydroxyethylbenzene (trimethylsylilether) of **5a**.

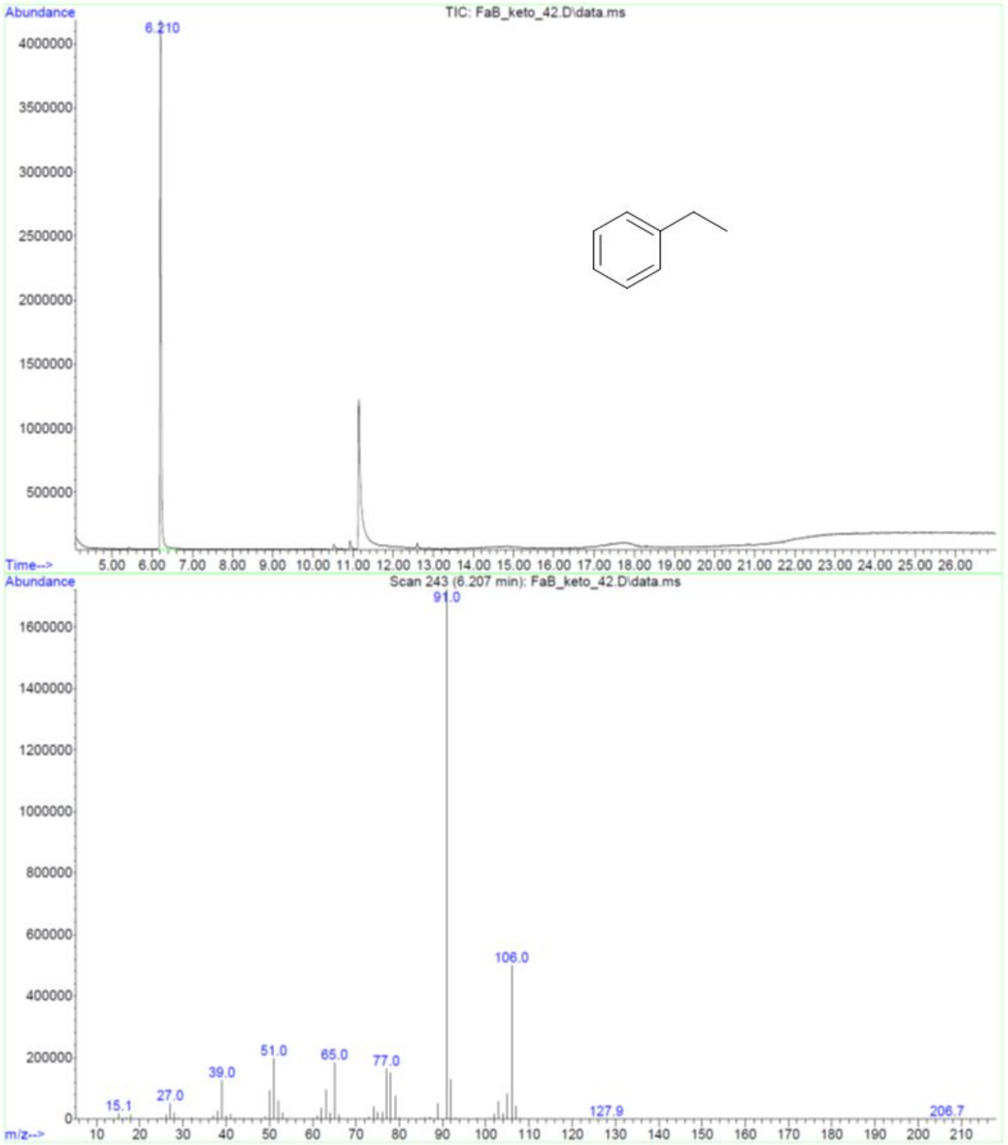

Figure S17: GC-MS identification of ethylbenzene **5b**.

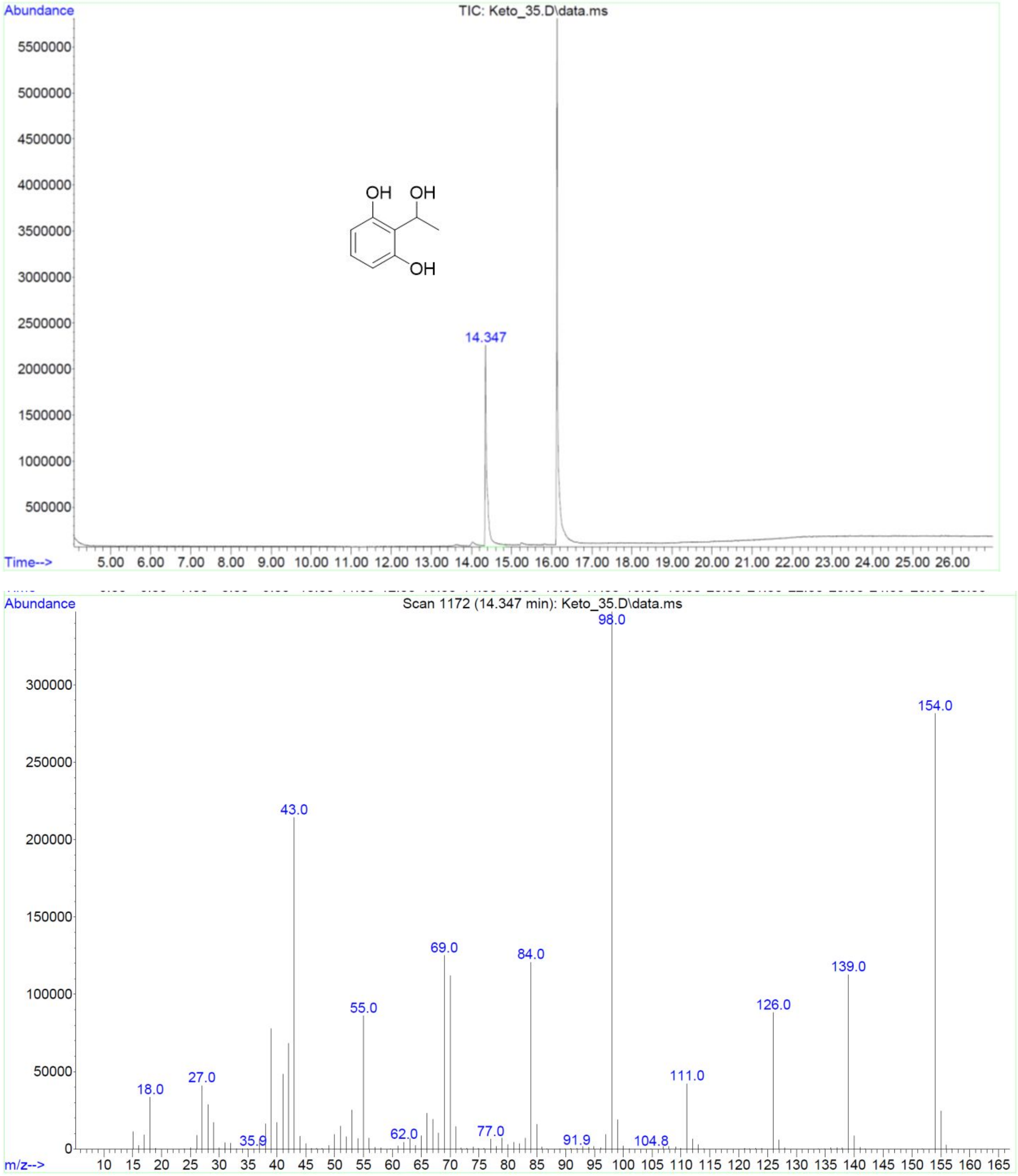

Figure S18: GC-MS identification of **2,6-dihydroxyphenylethanol (3c)**. Retention time: 14.347 min. MW: 154. Observed m/z: 154, 139, 126, 111, 98, 84, 69, 55, 43, 27, 18.

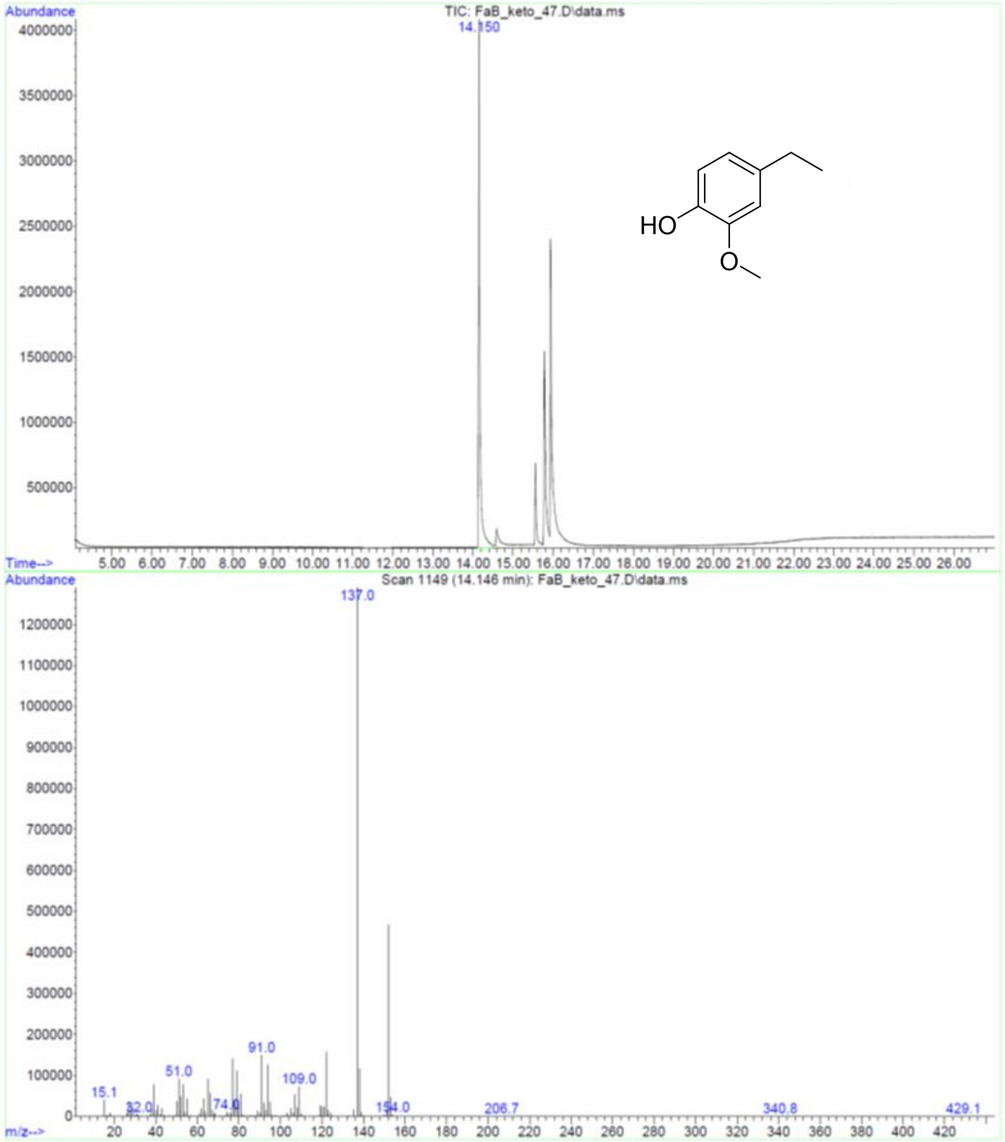

Figure S19: GC-MS identification of 4-ethyl-2-methoxyphenol **5d**.

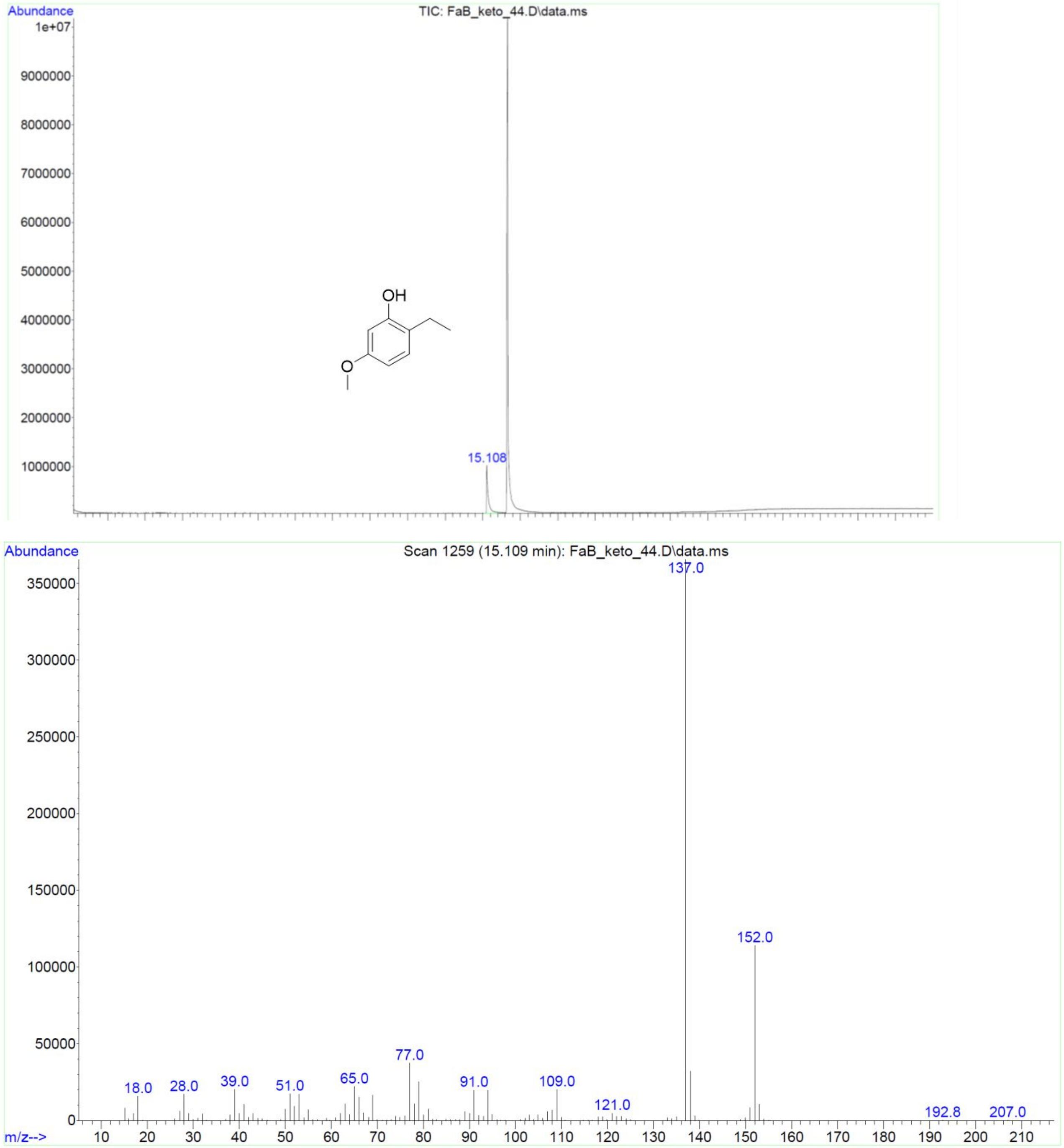

Figure S20: GC-MS identification of 2-hydroxy-4-methoxyethylbenzene **5e**.

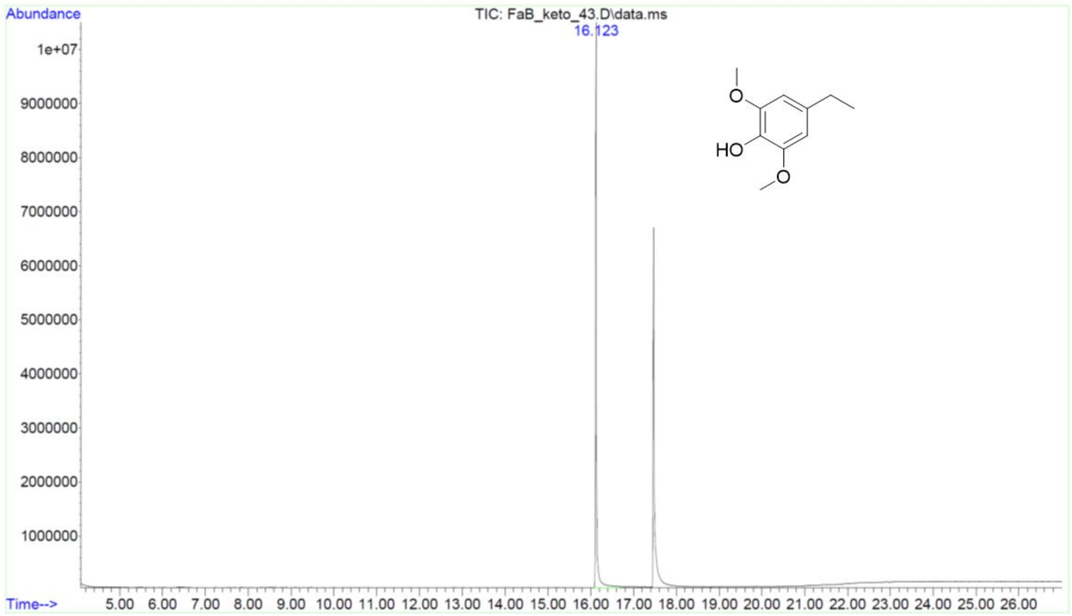

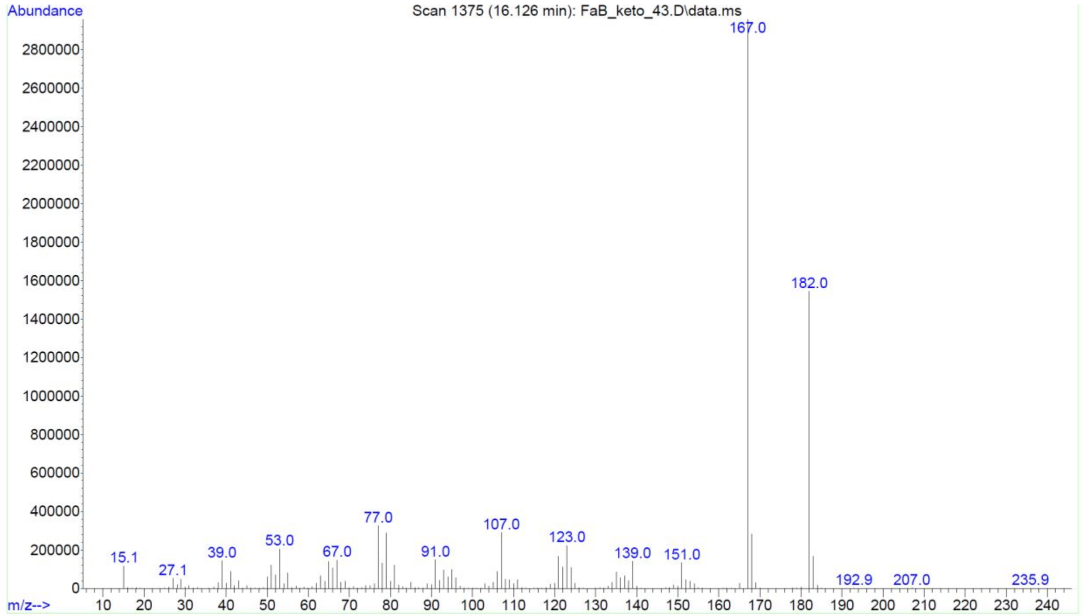

Figure S21: GC-MS identification of 4-hydroxy-3,5-dimethoxyethylbenzene **5f**.

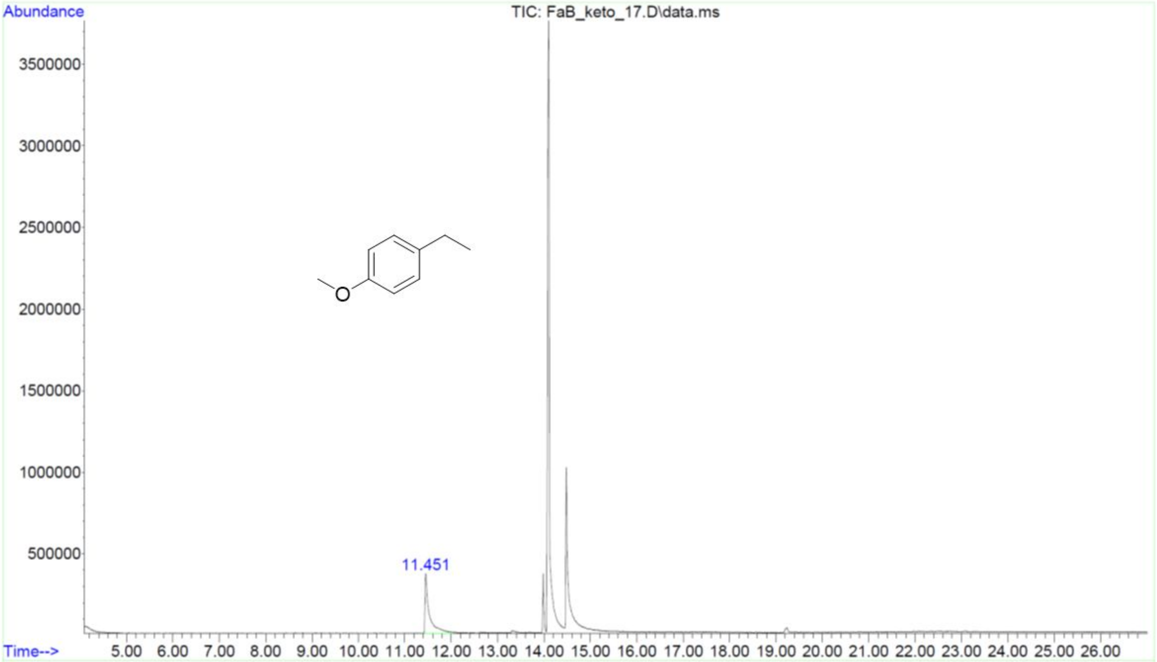

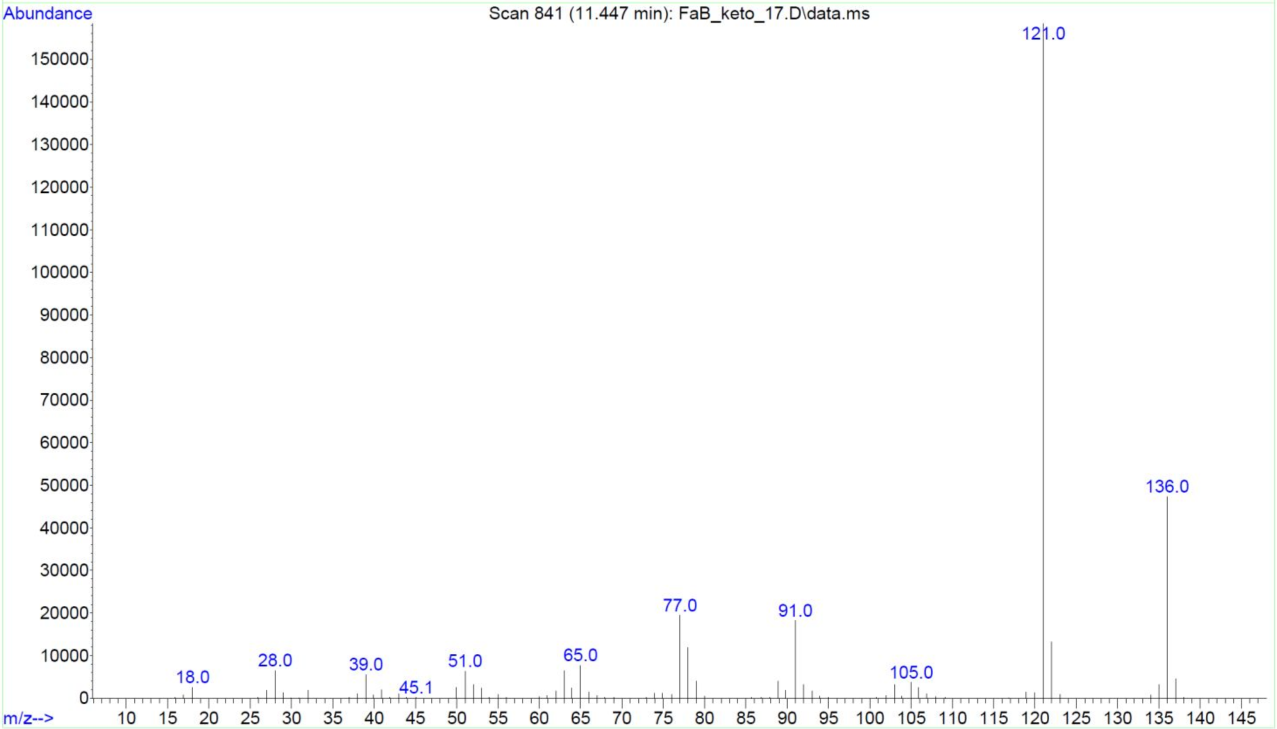

Figure S22: GC-MS identification of 4-methoxyethylbenzene **5g**.

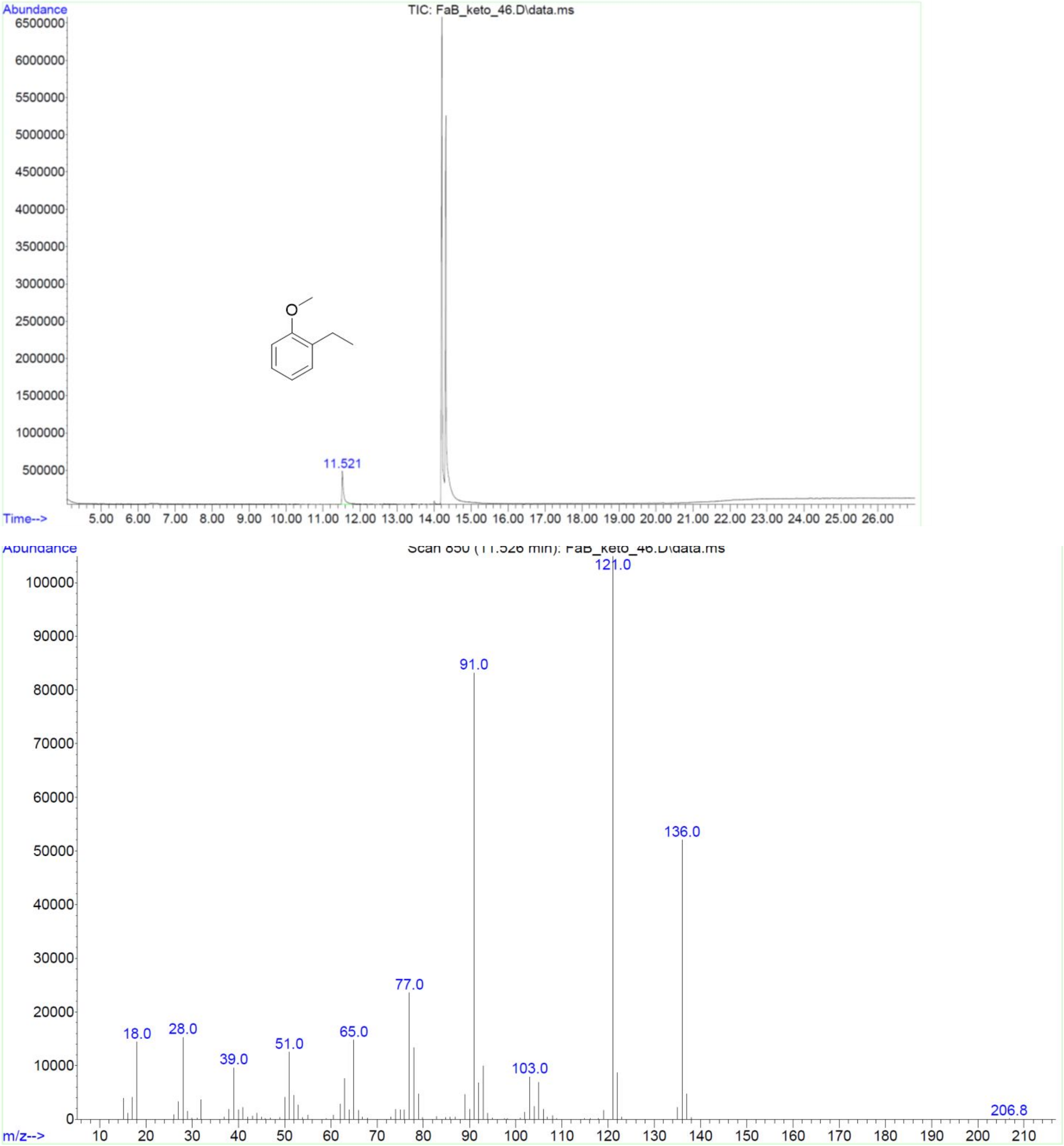

Figure S23: GC-MS identification of 2-methoxyethylbenzene **5h**. Retention time: 11.521 min. MW: 136. Observed m/z: 136, 121, 103, 91. 77. 65. 51. 39, 28, 18.

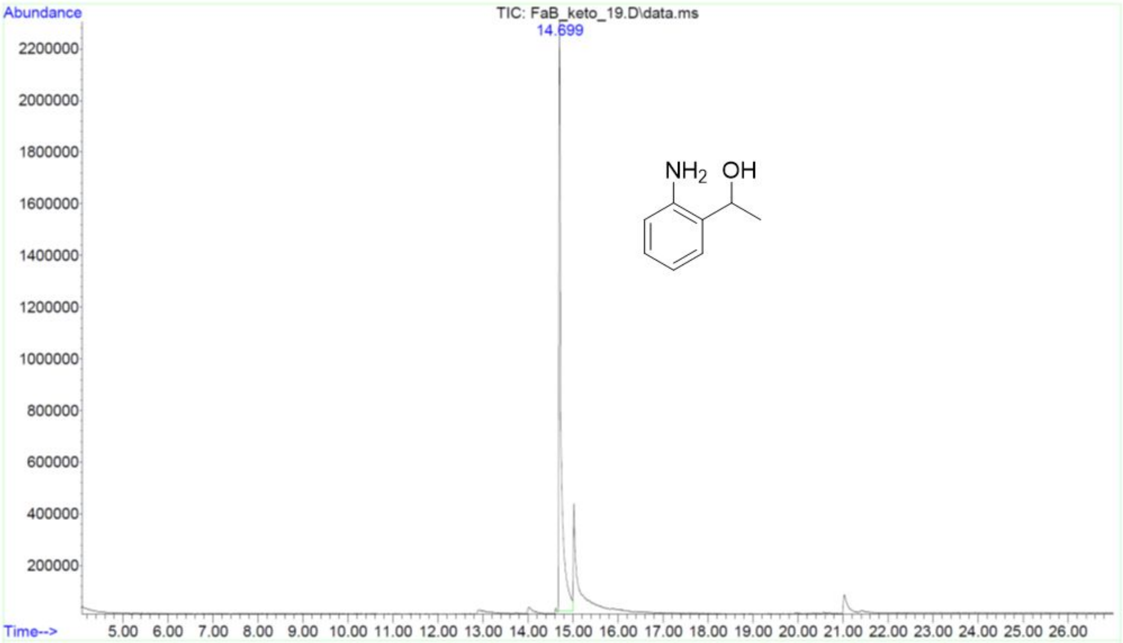

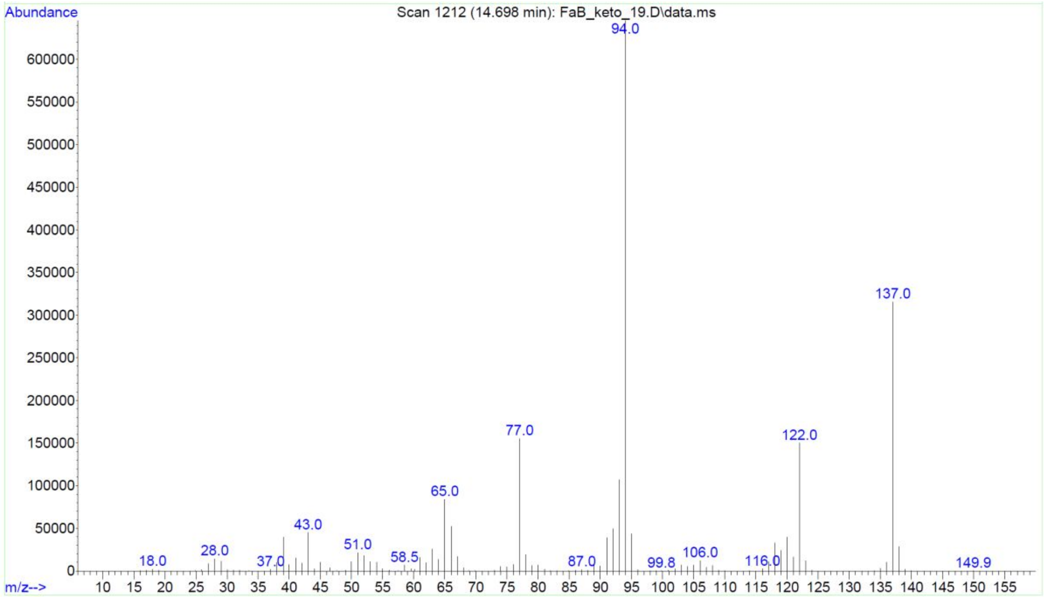

Figure S24: GC-MS identification of 1-(2-aminophenyl)ethanol. Retention time: 14.699 min. MW: 137. Observed m/z: 137, 122, 94, 77, 65, 51, 43, 28, 18.

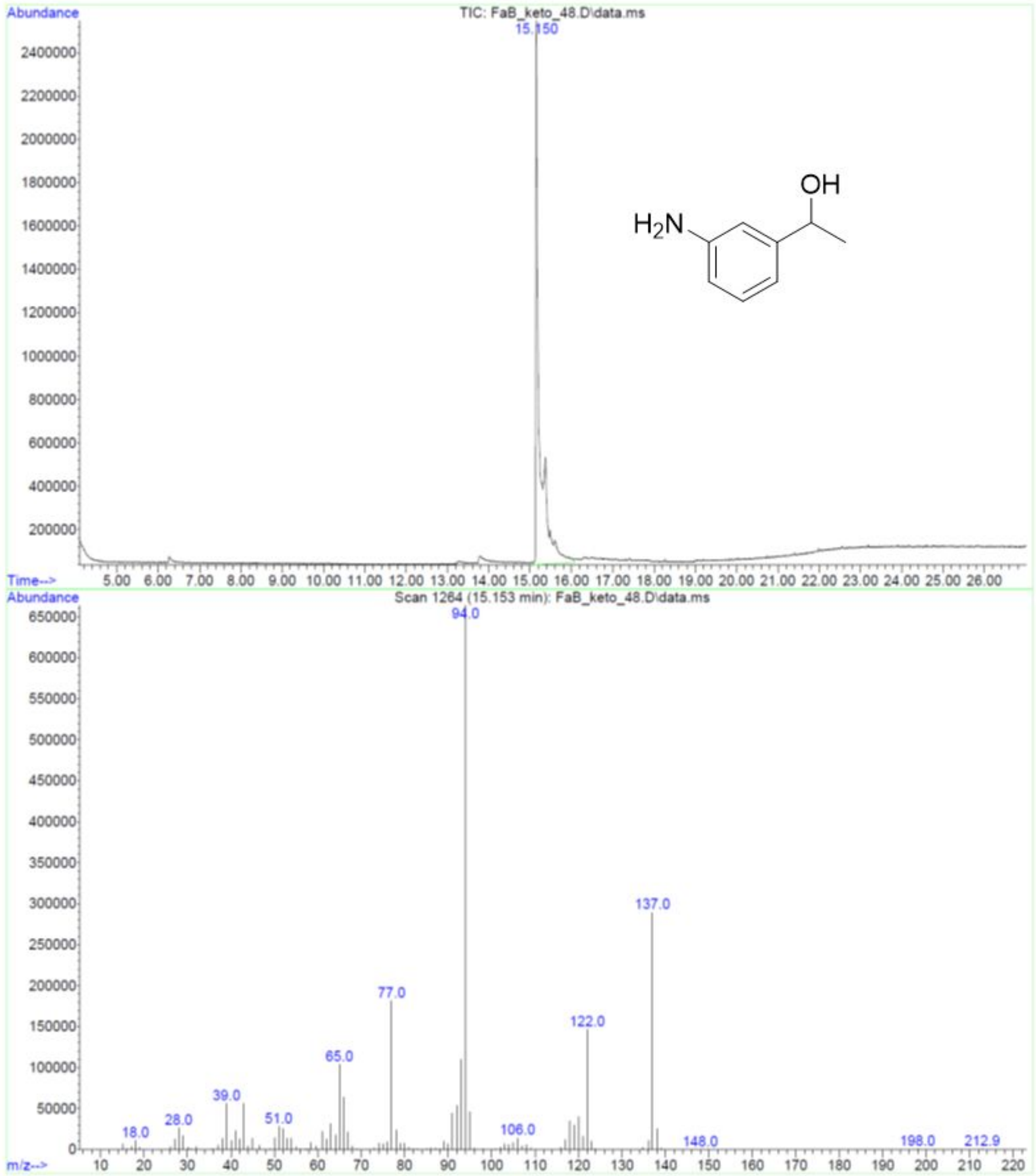

Figure S25: GC-MS identification of 3-aminophenylethanol. Retention time: 15.150 min. MW: 137. Observed m/z: 137, 122, 105, 94, 77, 65, 51, 43, 28, 18.

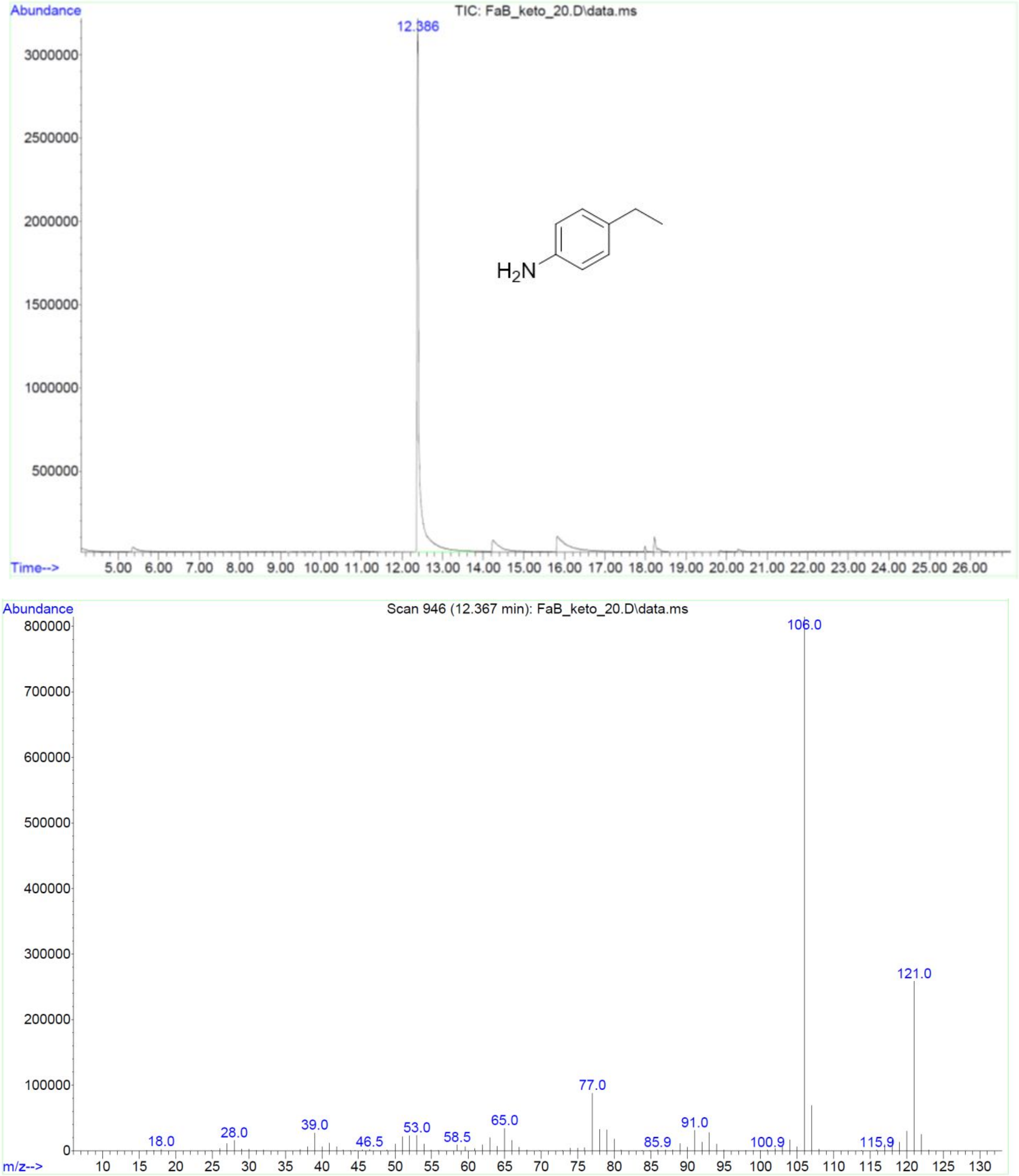

Figure S26: GC-MS identification of 4-aminoethylbenzene **5l**.

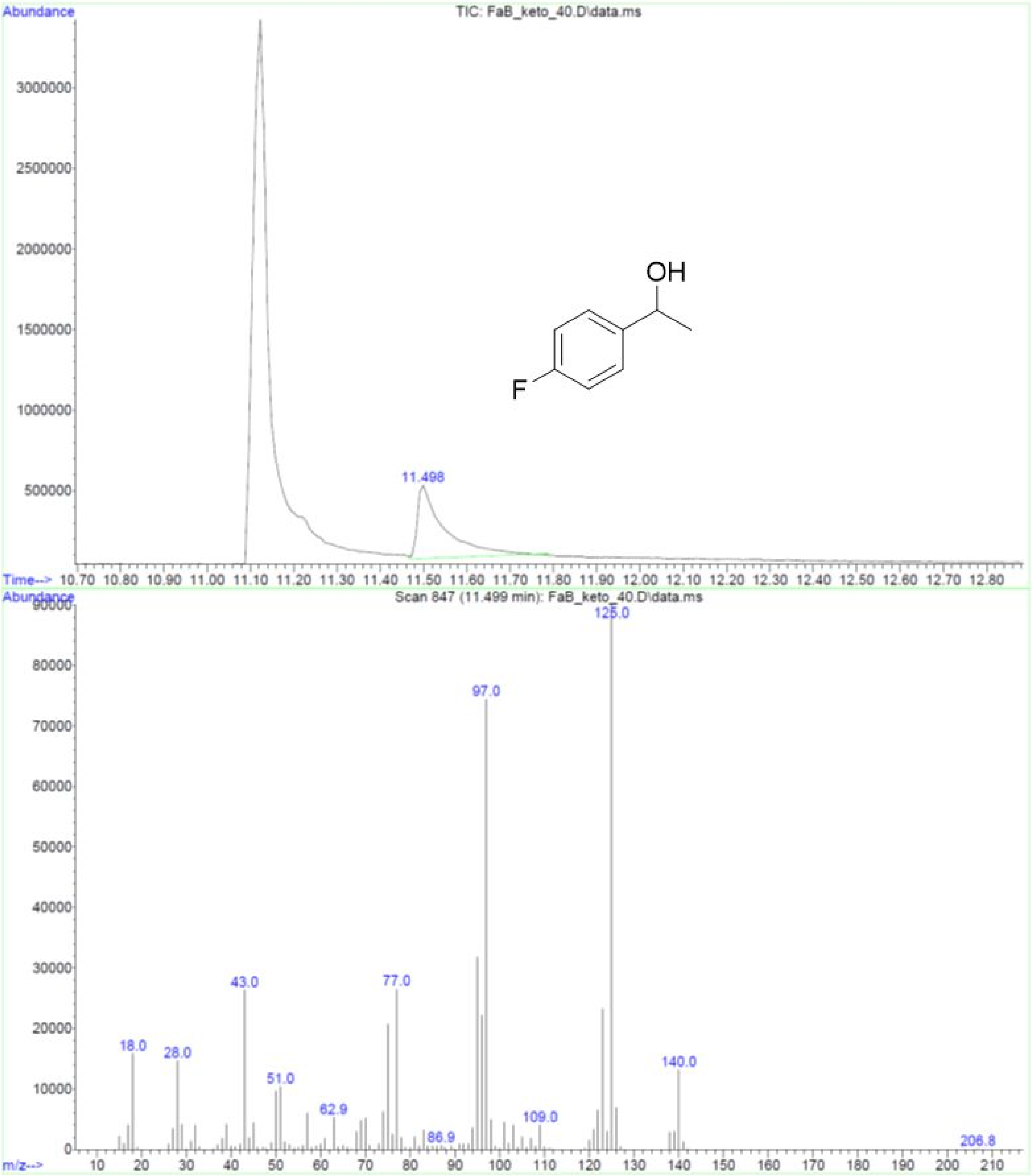

Figure S27: GC-MS identification of 4-fluorophenylethanol (**1l**). Retention time: 11.498 min. MW: 140. Observed m/z: 140, 125, 109, 97, 77, 63, 51, 43, 28, 18.

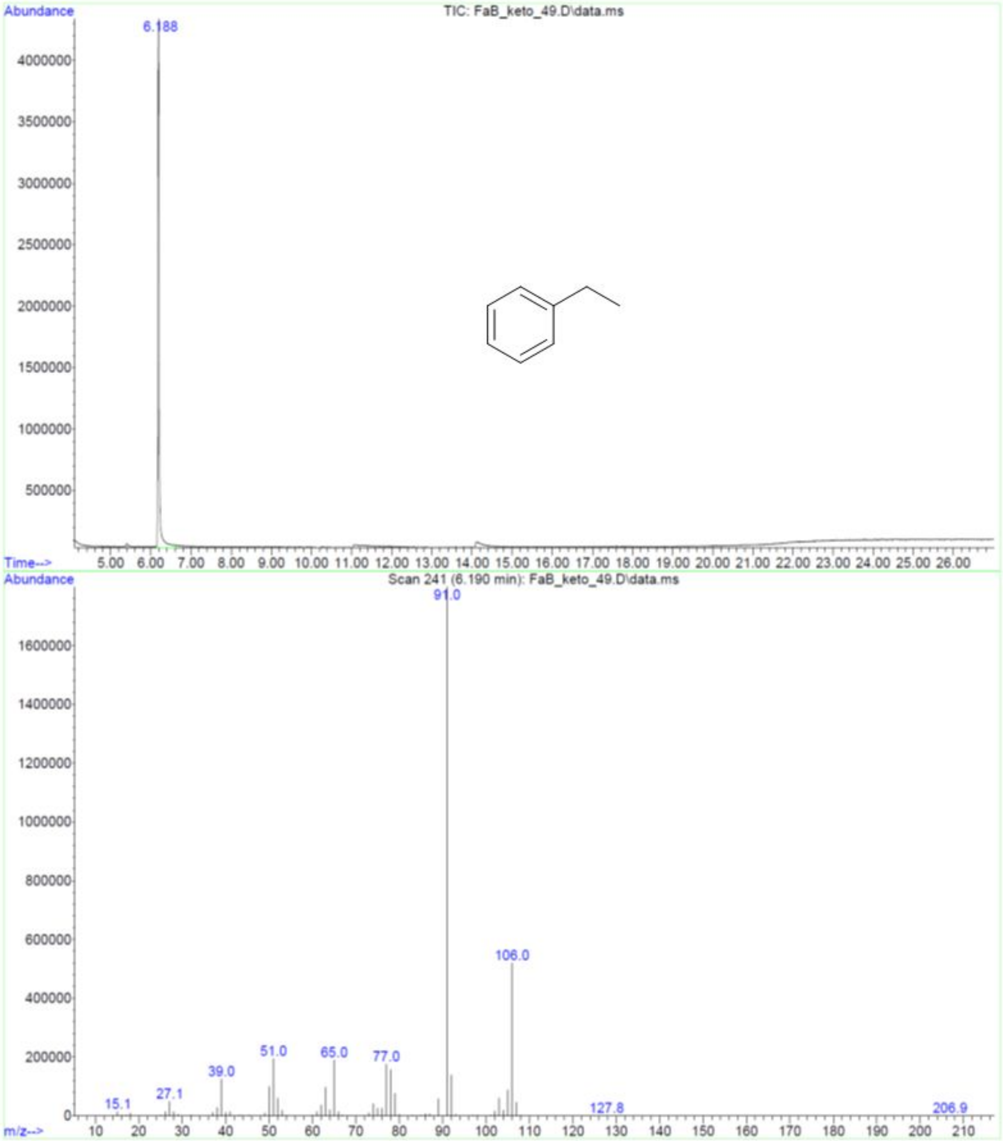

Figure S28: GC-MS identification of ethylbenzene from the reduction of 4-chloroacetophenone (**1m**). Retention time: 6.188 min. MW: 106. Observed m/z: 106, 91, 77, 65, 51, 39, 27, 15.

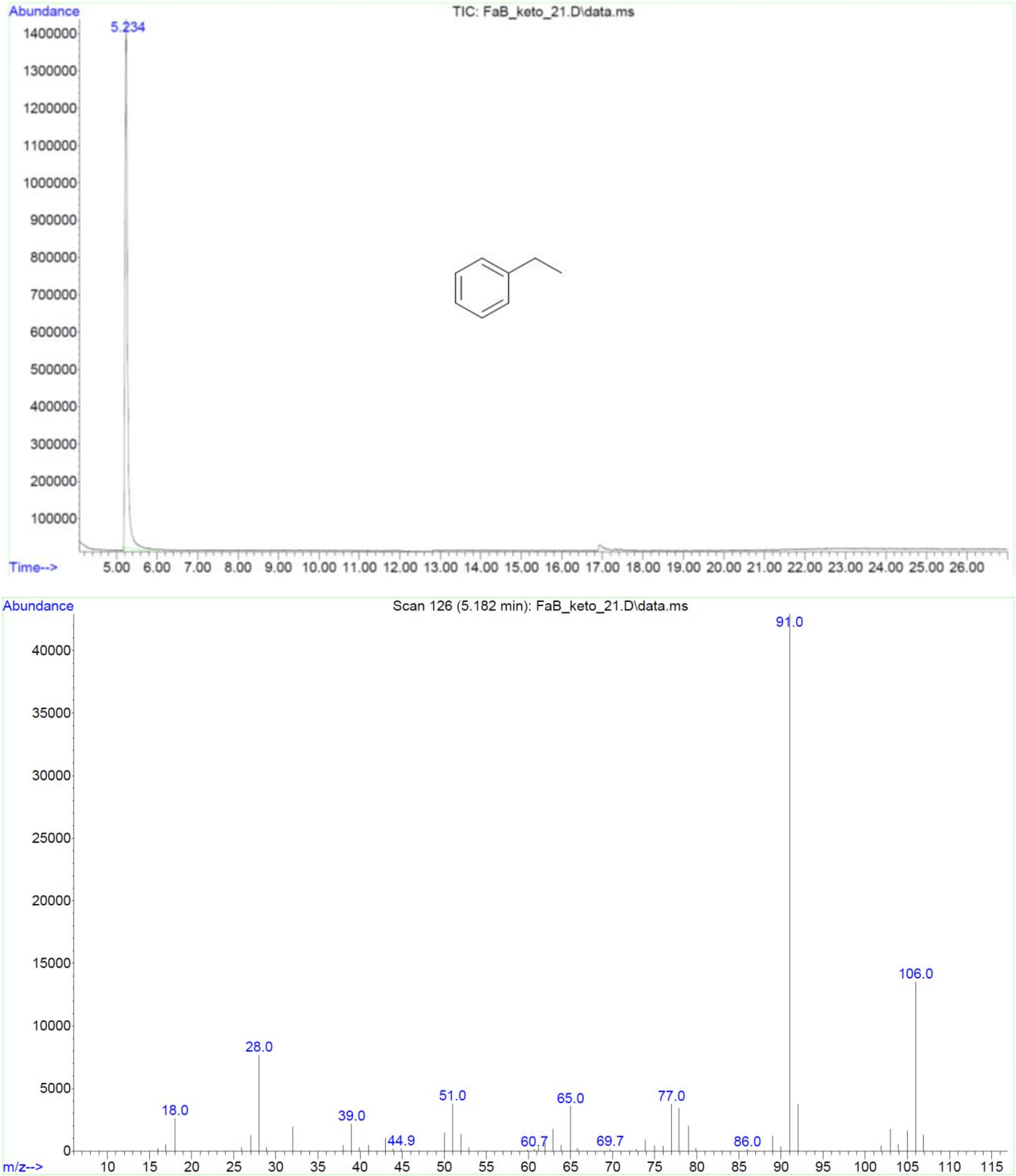

Figure S29: GC-MS identification of ethylbenzene from the reduction of 4-bromoacetophenone (**1n**). Retention time: 5.234 min. MW: 106. Observed m/z: 106, 91, 77, 65, 51, 39, 28, 18.

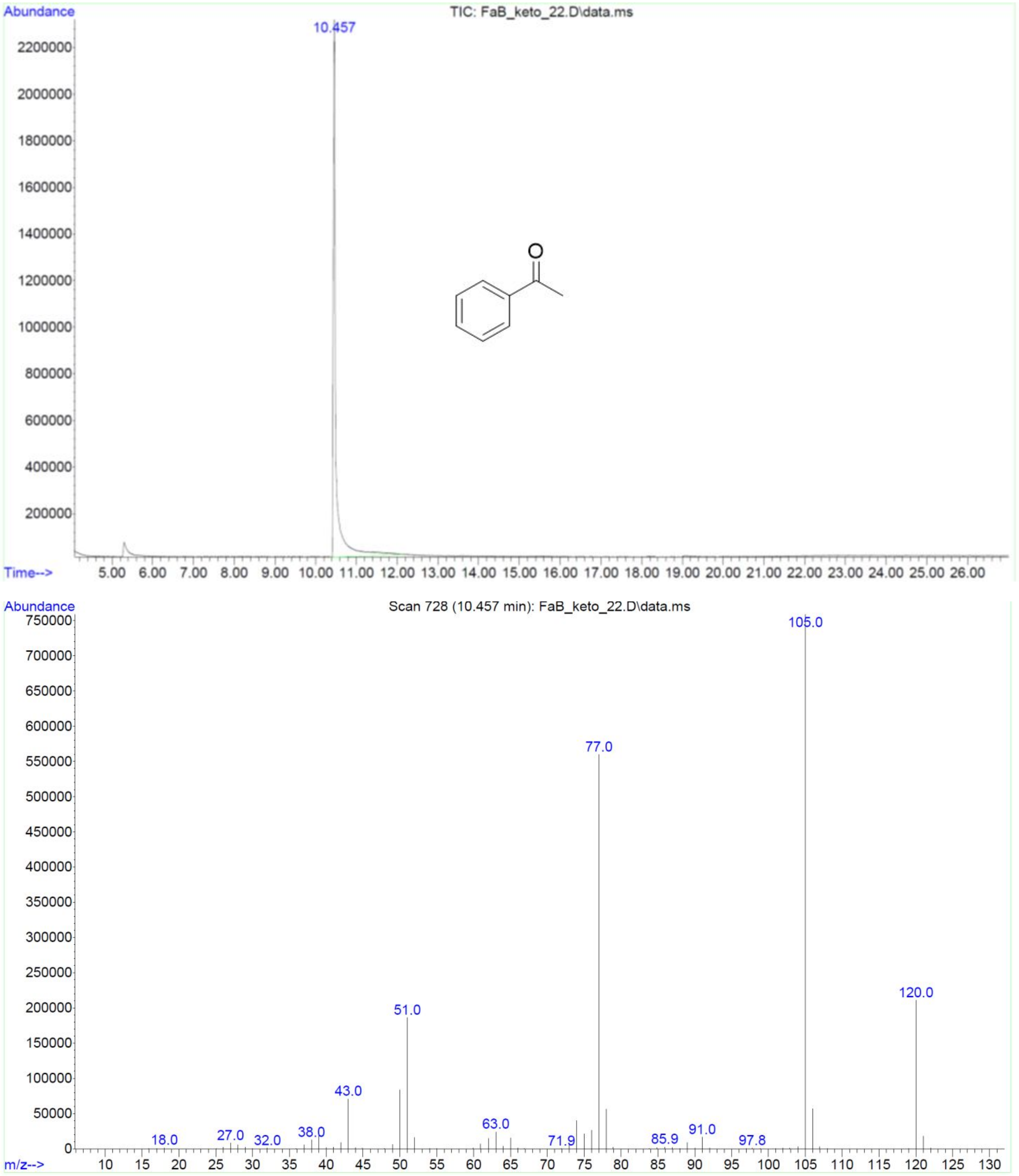

Figure S30: GC-MS identification of acetophenone from the reduction of 2-bromoacetophenone (**1o**). Retention time: 10.457 min. MW: 120. Observed m/z: 120, 105, 91, 86, 77, 63, 51, 43, 38, 27, 18.

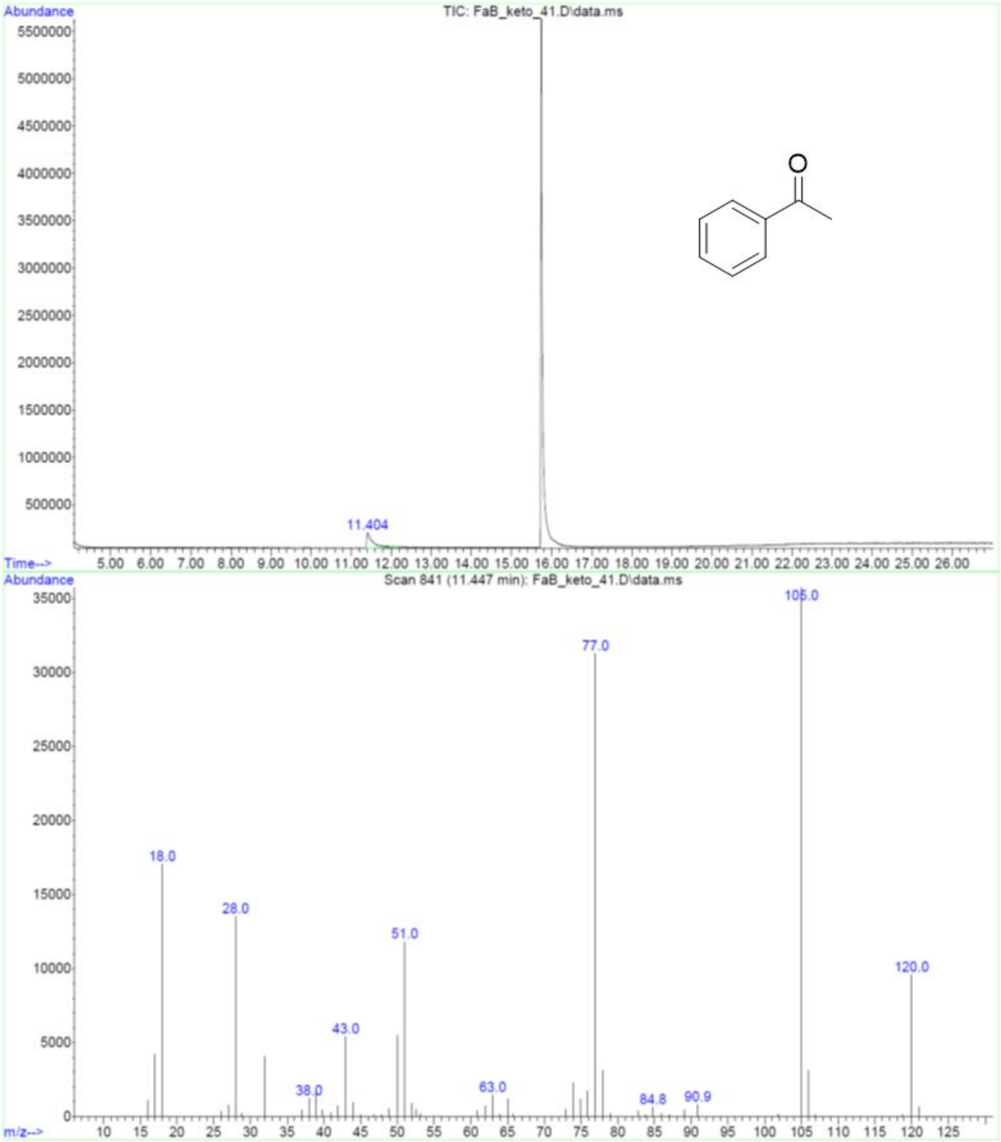

Figure S31: GC-MS identification of acetophenone from the reduction of 4-iodoacetophenone (**1p**). Retention time: 11.404 min. MW: 120. Observed m/z: 120, 105, 91, 85, 77, 63, 51, 43, 38, 27, 18.

Bibliography

1. Gao, S.-Y., Tang, Y.-Y.; † Yang, L. Zhang, P-l. Direct Hydrodecarboxylation of Carboxylic Acids via N-Hydroxyphthalimide-Mediated Hydrogen-Atom Transfer. *J. Org. Chem*. **2025**, *90*, 23, 7923–7929.
2. Lu, Y.-C.; West, J. G. Chemoselective Decarboxylative Protonation Enabled by Cooperative Earth-Abundant Element Catalysis. *Angew. Chem. Int. Ed*. **2023**, *62*, e202213055; *Angew. Chem*. **2023**, *135*, e202213055.
3. Zhou, X.; Yu, T.; Dong, G. Site-Specific and Degree-Controlled Alkyl Deuteration via Cu-Catalyzed Redox-Neutral Deacylation. *JACS*, **2022** *144* (22), 9570-9575.
4. Biswal, P.; Kumar, G.S.; Chandrasekhar, V. AgSbF6 Catalyzed Reduction of Nitroarenes by Phenylsilane to Anilines. *J. Org. Chem.* **2025** *90 (9)*, 3194-3201.
